# Cytological observation and transcriptome analysis reveal dynamic changes of *Rhizoctonia solani* colonization on leaf sheath and different genes recruited between the resistant and susceptible genotypes in rice

**DOI:** 10.3389/fpls.2022.1055277

**Published:** 2022-11-03

**Authors:** Sanglin Liu, Tianya Wang, Guoxian Meng, Jiahao Liu, Dibai Lu, Xiangdong Liu, Yuxiang Zeng

**Affiliations:** ^1^ State Key Laboratory for Conservation and Utilization of Subtropical Agro-Bioresources, South China Agricultural University, Guangzhou, China; ^2^ Guangdong Laboratory for Lingnan Modern Agriculture, South China Agricultural University, Guangzhou, China; ^3^ Guangdong Provincial Key Laboratory of Plant Molecular Breeding, College of Agriculture, South China Agricultural University, Guangzhou, China; ^4^ State Key Laboratory of Rice Biology, China National Rice Research Institute, Hangzhou, China

**Keywords:** rice, *Rhizoctonia solani*, sheath blight, RNA-seq, confocal microscopy

## Abstract

Sheath blight, caused by *Rhizoctonia solani*, is a big threat to the global rice production. To characterize the early development of *R. solani* on rice leaf and leaf sheath, two genotypes, GD66 (a resistant genotype) and Lemont (a susceptible genotype), were observed using four cytological techniques: the whole-mount eosin B-staining confocal laser scanning microscopy (WE-CLSM), stereoscopy, fluorescence microscopy, and plastic semi-thin sectioning after *in vitro* inoculation. WE-CLSM observation showed that, at 12 h post-inoculation (hpi), the amount of hyphae increased dramatically on leaf and sheath surface, the infection cushions occurred and maintained at a huge number from about 18 to 36 hpi, and then the infection cushions disappeared gradually from about 42 to 72 hpi. Interestingly, *R. solani* could not only colonize on the abaxial surfaces of leaf sheath but also invade the paraxial side of the leaf sheath, which shows a different behavior from that of leaf. RNA sequencing detected 6,234 differentially expressed genes (DEGs) for Lemont and 7,784 DEGs for GD66 at 24 hpi, and 2,523 DEGs for Lemont and 2,719 DEGs for GD66 at 48 hpi, suggesting that GD66 is recruiting more genes in fighting against the pathogen. Among DEGs, resistant genes, such as *OsRLCK5*, *Xa21*, and *Pid2*, displayed higher expression in the resistant genotype than the susceptible genotype at both 24 and 48 hpi, which were validated by quantitative reverse transcription–PCR. Our results indicated that the resistance phenotype of GD66 was the consequence of recruiting a series of resistance genes involved in different regulatory pathways. WE-CLSM is a powerful technique for uncovering the mechanism of *R. solani* invading rice and for detecting rice sheath blight–resistant germplasm.

## Introduction

Rice sheath blight, caused by *Rhizoctonia solani*, is a serious disease in many rice-producing countries (Zeng et al., 2017). The yield losses caused by sheath blight have exceeded other major diseases, making it the most serious rice disease in China (Zeng et al., 2011). Sheath blight caused about 20% yield losses in India, and it is also the most common rice disease in the Southern United States (Groth and Bond, 2007; Ghosh et al., 2016).

The soilborne fungal pathogen *R. solani* infects more than 250 plant species (Anderson, 1982). Plants immune to this pathogen have not been found. All the rice plants can be infected by this pathogen, but the serious extent varies among different cultivars. Large-scale screening in rice communities have found some rice varieties that showed partial resistance, such as Teqing (Li et al., 1995), Jasmine 85 (Pan et al., 1999; Zou et al., 2000; Park et al., 2008), Tetep (Channamallikarjuna et al., 2010), WSS2 (Sato et al., 2004), Pecos (Sharma et al., 2009), ARC10531 (Yadav et al., 2015), and GD66 (Zeng et al., 2017). Partial resistance means that they perform better resistance than other varieties, although none of which are immune to this pathogen.

Fungal pathogens usually produce some infection structures to invade and establish parasitic relationship with the host plant. The infection structures, such as infection cushion (Dodman et al., 1968; Dodman and Flentje, 1970), appressoria (Emmett and Parbery, 1975), infection peg, and haustorium (Riopel and Timko, 1995; Huang et al., 2003), are formed by specialized hyphae. Although there is still much debate about the details of the infection process, most researchers now agree that *R. solani* hyphae invade rice mainly in two ways: (1) Hyphae gather to form two specialized infection structures: infection cushion and appressoria on the surface of leaf sheath (Marshall, 1980a; Deising et al., 2000). The infection cushion may be conducive to the concentration of sheath blight pathogen energy and nutrition to infect the host and to destroy the plant defense system and obtain more nutrition and water from the host cells for the mass reproduction of the pathogen (Zhang et al., 2010). There are invasion nails and holes at the base of the contact surface between the infection cushion and the rice epidermis. After the invasive nail contacts the surface of leaf sheath, it secretes mucilaginous substances, which can poison plants (Matsuura, 1986). The infected nail became obviously thinner when invading epidermal cells, and it returned to its original diameter after entering cells (Tang, 2011). The mycelium expands and covers one side of the cell wall, it becomes very thin to pass through the cell wall, and then widens to restore the mycelium size (Zhang et al., 2010). (2) Hyphae can directly penetrate into rice epidermis without invasive structure. It penetrates rice epidermal cells and the wall between cells, invades rice leaf sheath from intercellular space, or directly invades host from natural orifice (mainly stomata) and wound (Marshall, 1980b; Tao, 1992; Zhang et al., 2010; Tang, 2011). Therefore, the infection structure is not a necessary device for penetration. Hyphae can form penetrating claws that exert great invasive pressure on rice epidermal cells to enter the host (Zhang et al., 2010; Tang, 2011). The primary hyphae form secondary hyphae after entering the leaf sheath, which grow in the host or expand in the intercellular space (Zhang et al., 2010). In addition, the mycelium is very thick and deformed in rice cells (Singh et al., 2003).

Genetic analysis revealed that sheath blight resistance in rice is controlled by quantitative trait loci (QTLs) with minor effects (Kump et al., 2011). In addition, the resistance is conferred by the additive effect of non–race-specific resistance QTLs (Pinson et al., 2005; Liu et al., 2009). Approximately 50 sheath blight–resistant QTLs have been detected on all the 12 rice chromosomes (Zuo et al., 2010; Xu et al., 2011), and QTL by environment interaction is frequently detected. More than 20 genes conferring sheath blight resistance have been cloned and characterized, including genes that positively regulate sheath blight resistance: *OsWRKY4* (Wang et al., 2015a) *OsWRKY13* (John and Subramanian, 2019), *DOF11* (Kim et al., 2021), *OsSWEET14* (Kim et al., 2021), *OsPGIP1* (Wang et al., 2015b), *OsRSR1* (Wang et al., 2021), *OsGLP1* (Banerjee and Maiti, 2010), *Os2H16* (Li et al., 2013), *IDD13* (Sun et al., 2020), *OsOSM1* (Xue et al., 2016), *OsRLCK5* (Wang et al., 2021), *OsWAK25* (Harkenrider et al., 2016), *LPAl* (Sun et al., 2019), *OsPAL4* (Tonnessen et al., 2015), and *OsCHI11* (Karmakar et al., 2016). Genes that negatively regulated sheath blight resistance were also characterized: *OsWRKY45* (Shimono et al., 2012), *OsWRKY53* (Yuan et al., 2020), *OsSWEET11* (Gao et al., 2018), *OsTrxm* (Hu et al., 2021), *IDD3* (Sun et al., 2020), *OsNYC3* (Cao et al., 2022), *OsGFl4e* (Manosalva et al., 2011), *OsMESL* (Hu et al., 2021), *OsBON1* (Yin et al., 2018), and *OsAKT1* (Yuan et al., 2020). These sheath blight–resistant genes participated in different regulatory pathways, including reactive oxygen species (ROS), phytohormone, salicylic acid signaling, defense response to fungi and innate immune, sucrose transport, and other regulatory pathways.

Transcriptome analysis by RNA sequencing (RNA-seq) is a powerful tool to measure the spatial and temporal expression of various genes in response to outer stimulus and to demonstrate from the macroscopic view that different genes participated in different biological pathways (Słomnicka et al., 2021). The transcriptome profiles between the susceptible cultivar Lemont and the moderately resistant cultivar Teqing in response to sheath blight disease were studied, and it showed that regulation of photosynthesis, photorespiration, jasmonic acid, and phenylpropanoid pathways contribute to rice resistance to *R. solani* (Shu et al., 2019). Transcriptome analysis showed that the defense system in resistant rice cultivars involved the expression of *PR* genes, key transcription factors, *PAL* genes, and the enrichment of defense-related pathways (Yang et al., 2022). Comparative transcriptome analysis suggested that photosynthesis, photorespiration, and jasmonic acid and phenylpropanoid metabolism played important roles in disease resistance (Zhang et al., 2017).

Despite great efforts made in the rice sheath blight research communities, there are still many questions that need to be addressed: What is the detailed developmental progress of the *R. solani* hyphae on rice leaf and leaf sheath at different time spots under both fluorescence microscope and confocal microscope? Are there any differences between sheath blight–resistant and sheath blight–susceptible varieties during the hyphal growth? To breed a sheath blight–resistant cultivar, what can we learn from the transcriptome analysis results? To answer these questions, we first observed the hyphal growth on rice leaf and leaf sheath using a stereoscope, a fluorescence microscope, and the whole-mount eosin B-staining confocal laser scanning microscopy (WE-CLSM) to gain an overall picture of how the fungal hyphae were grown and spread on leaf and leaf sheath. We demonstrate that WE-CLSM can be used conveniently to observe the *R. solani* hyphae inside rice leaf or leaf sheath. This new method provides rapid observation of the cytological progress of *R. solani* hyphae invading rice that would benefit sheath blight studies in the future. Moreover, transcriptome analysis between a sheath blight–resistant genotype GD66 and a sheath blight–susceptible genotype Lemont was studied after pathogen inoculation to reveal the sheath blight–resistant mechanism and to discuss how to breed an ideal cultivar with good sheath blight resistance.

## Materials and methods

### Rice genotypes

Five rice genotypes (Lemont, Taichung65, Yinhesizhan, 8821, and GD66) were used in the present study. Lemont, Yinhesizhan, and GD66 were provided by the National Midterm Rice Genebank in China National Rice Research Institute (CNRRI). Taichung65 and 8821 were provided by the State Key Laboratory for Conservation and Utilization of Subtropical Agro-Bioresources, South China Agricultural University (SCAU). Lemont is a *japonica* American cultivar. Taichung65 is a *japonica* cultivar originated from Taiwan, China. Yinhesizhan is an *indica* cultivar originated from Guangdong, China. 8821 is an *indica* cultivar developed by Zhaoqing Institute of Agricultural Sciences, Guangdong, China. GD66 is an *indica* restorer line developed by CNRRI. These genotypes were grown under a natural light in a greenhouse with the temperature ranged from 20°C to 35°C or in the field with common practices in South China Agricultural University (23.16N, 113.35E), Guangzhou, China.

### *R. solani* isolate for inoculation

The *R. solani* isolate ZJ03, collected in Fuyang, Hangzhou, China, was used for inoculation in this study. The isolate ZJ03 has been used for testing rice sheath blight resistance and QTL mapping in some previous studies (Zeng et al., 2017; Chen et al., 2019; Zeng et al., 2021).

### Inoculation and evaluation of sheath blight resistance in the field

The rice genotypes were sown on 25 February 2021 in the farm of SCAU, in Guangzhou, China. Fifty individual plants were planted in a plot for each genotype. The 50 plants were arranged in five rows with 10 plants in each row. The space between each row and different plants within each row were both 10 cm. At the late tillering stage, five plants in the middle of the third row were inoculated using ZJ03. A total of 10 tillers were inoculated for each genotype, i.e., two tillers for each individual plant. The inoculation method were described by Zou et al. (2000) and Zeng et al. (2017): toothpicks covering with mycelium were used to penetrate the third leaf sheath, counting from the top. Sheath blight resistance was evaluated 30 days after inoculation. The lesion length was measured vertically along the stem for each genotype. The visual rating system of 0 to 9 was used to evaluate each inoculated individual, where 0 indicated that the plant was immune to the pathogen and 9 indicated a dead or collapsed plant (Pinson et al., 2005).

### *In vitro* inoculation

At the booting stage, the second leaf sheath counting from the top and the leaf that connected to the sheath were scissored off the plant and washed using tap water for surface cleaning. Then, the sheath and leaf were cut into uniform lengths of 10 cm from the middle and placed in the plastic Petri dish of 13 × 13 cm covered with two layers of sterile gauze according to the method described by Vijay et al. (2011) with some modifications: Three leaves or sheaths were placed in each Petri dish, and the bottom side of the leaf or sheath was covered with gauze. Three replications, i.e., nine leaves or sheaths in a total of three Petri dishes, were performed to ensure consistent and reliable results. ddH_2_O (20 ml) was added to each Petri dish to keep the gauze moisty. The *R. solani* isolate ZJ03 was cultured and maintained in the potato dextrose agar (PDA) medium at 28°C in a growth chamber. First, the ZJ03 sclerotia or mycelia were transferred into a new PDA medium. In about 3 days, the pathogen mycelia occupied the entire surface of the PDA medium. Then, a 5-mm-diameter PDA medium covered with mycelia was placed in the middle of the leaf or leaf sheath in the Petri dish (**Figure S1**). Finally, the Petri dishes were sealed with breathable sealing film and incubated in a growth chamber at 28 ± 1°C under a 16-h light/8-h dark condition. Sterile agar medium without pathogen mycelia was also placed on leaf or sheath surface as the control.

### Fluorescence microscopy

At the booting stage, the second leaf sheath counting from the top and the leaf connected to the sheath were inoculated *in vitro* in Petri dishes as described above. About 1.5-cm^2^ leaf (or sheath) area at the inoculation site was observed at different time spots after inoculation under a microscope. At the time spot of every 4 hpi (**Figures S2**, **S3**), the PDA medium covered with fungal was removed from the leaf (or sheath) surface and observed using a stereoscope (Leica MZ16, Germany) with 2× lens. After stereoscopic observation, the materials were directly stained with 1% 4',6-diamidino-2-phenylindole (DAPI) solution for 2 min and observed under a fluorescence microscope (Leica DMRXA, Germany).

### The whole-mount eosin B-staining confocal laser scanning microscopy

The WE-CLSM was applied to observe the mycelial growth of sheath blight pathogen following the previous protocol with minor modifications (Zeng et al., 2007): At the booting stage, rice leaf or sheath was collected every 6 h after *in vitro* inoculation and fixed in a formaldehyde–acetic acid–ethanol solution (70% ethanol:acetic acid:formaldehyde = 18:1:1, v/v) for at least 48 h. Then, the samples were washed with 50% ethanol and kept in 70% ethanol at 4°C. The samples were hydrated consecutively in 50%, 30%, and 10% ethanol and distilled water for 30 min at each concentration, respectively. Then, the samples were stained in eosin B solution (10 mg/L; dissolved in 4% sucrose solution) for 48 h. Finally, the samples were dehydrated sequentially in 10%, 30%, 50%, 70%, 90%, and 100% alcohol solution for 30 min at each concentration, respectively. The dehydrated samples were stored in 50% methyl salicylate (dissolved in pure alcohol) for 48 h. Finally, the samples were stored in pure methyl salicylate for 48 h and then observed under a Leica SPE laser scanning confocal microscope (Leica Microsystems, Heidelberg, Germany). Excitation wavelength was 543 nm, and emission wavelength was between 550 and 630 nm (Zeng et al., 2007). The leaf area about 0.5 cm^2^ at the inoculation position was observed under a confocal microscope.

### Transcriptome analysis

Lemont (a sheath blight–susceptible cultivar) and GD66 (a sheath blight–resistant restorer line) were used for transcriptome analysis by RNA-seq. The young seedlings of these two genotypes were first grown in green house. At the late tillering stage, the plants were transferred into a growth chamber (DHP-9052, Shanghai, China, 28°C, 16-h light/8-h dark condition) and cultured for 10 days before inoculation. The toothpicks covering with mycelia were used to penetrate the sheath for inoculation using the method described by Zou et al. (2000) and Zeng et al. (2017).

Total RNAs were extracted from inoculated sheath at 24 and 48 h post-inoculation (hpi). Leaf sheath at the inoculation site was collected and stored in liquid nitrogen for RNA extraction. The total RNA of each sample was extracted using TRIzol reagent (Invitrogen, Waltham, MA, USA) following the manufacturer’s procedure. Transcriptome analyses were based on the results of three biological replicates.

RNA extraction, libraries construction, and sequencing were conducted on the basis of the manufacturer’s instructions of Illumina HiSeqTM2500 (LC Sciences, Hangzhou, China) by Biomarker Technologies (Beijing, China) (Yu et al., 2018). The differentially expressed genes (DEGs) were identified using DESeq software with the following criteria: fold change (FC) ≥ 2 and false discovery rate (FDR) ≤ 0.01. Genes with *p-*values < 0.05 were chosen for further analysis.

After selecting the DEGs, cluster analysis and Gene Ontology (GO) enrichment analysis were conducted using Cluster 3.0 software (Tokyo, Japan) and agriGO 2.0 (Beijing, China) (http://systemsbiology.cau.edu.cn/agriGOv2/SEAresult2.php), respectively. Venny 2.1 software (BioinfoGP, Madrid, Spanish) was used to identify the overlapped DEGs in different samples (http://bioinfogp.cnb.csic.es/tools/venny/). Heat map was drawn by TBtools (Chen et al., 2020). UpSet plot were delineated using an online OmicStudio tools (https://www.omicstudio.cn/tool). The MapMan software was downloaded from the Internet for MapMan analysis (https://mapman.gabipd.org).

### qRT-PCR analysis

A total of seven candidate genes were selected for validation using quantitative reverse transcription (qRT)–PCR. The gene-specific primers (**Table S1**) for qRT-PCR were designed using a Primer Premier 5.0 software. The total RNA was extracted by AG RNAex Pro Reagent and was reverse-transcribed into cDNA with the Evo M-MLV RT Kit (Accurate Biotechnology Co., Ltd., Hunan, China). All operations were implemented according to the kit instructions. The amplification reaction was performed on the Lightcycler480 system (Roche, Basel, Switzerland) using the Advanced SYBR Green Supermix Kit (Bio-Rad, Hercules, CA, USA). The total volume of reaction system was 20 μl. The qRT-PCR cycles were performed using the following reaction procedure: 95°C for 30 s, 40 cycles of 95°C denaturation for 5 s, and 58°C annealing and extension for 20 s. The relative expression level of genes was calculated using the 2^−ΔΔCt^ method (Livak and Schmittgen, 2013). The rice ubiquitin gene was used as an internal control to normalize the expression levels (Wu et al., 2014). All samples were conducted for three biological replications.

## Results

### Sheath blight resistance of different genotypes examined in the field

Five rice genotypes inoculated under the field condition showed different sheath blight resistance, with average lesion length ranging from 33.45 to 58.42 cm (**Table 1**). The sheath blight resistance of GD66 was significantly higher than that of Lemont in both lesion length and visual rating according to the Duncan’s multiple range test (**Table 1**, **Figure S4**).

**Table 1:** Evaluation of sheath blight resistance under the field condition.

Genotype	Lesion length (cm) mean ± SD	Visual rating mean ± SD	Resistance evaluation
Lemont	58.77 ± 2.38 c	7.26 ± 0.53 d	HS
Taichung65	47.86 ± 9.64 b	5.79 ± 1.26 c	MS
Yinhesizhan	42.39 ± 5.17 ab	5.14 ± 0.55 bc	MR
8821	38.71 ± 7.11 ab	4.49 ± 0.63 ab	HR
GD66	33.68 ± 6.76 a	3.95 ± 0.34 a	HR

HS, high sensitivity; MS, medium sensitivity; MR, medium resistance; HR, high resistance. Values in each column are significantly different (P = 0.05) if marked by different letters according to the Duncan’s multiple range test.

### Lesion on leaf surface observed after *in vitro* inoculation

**Figure 1:**
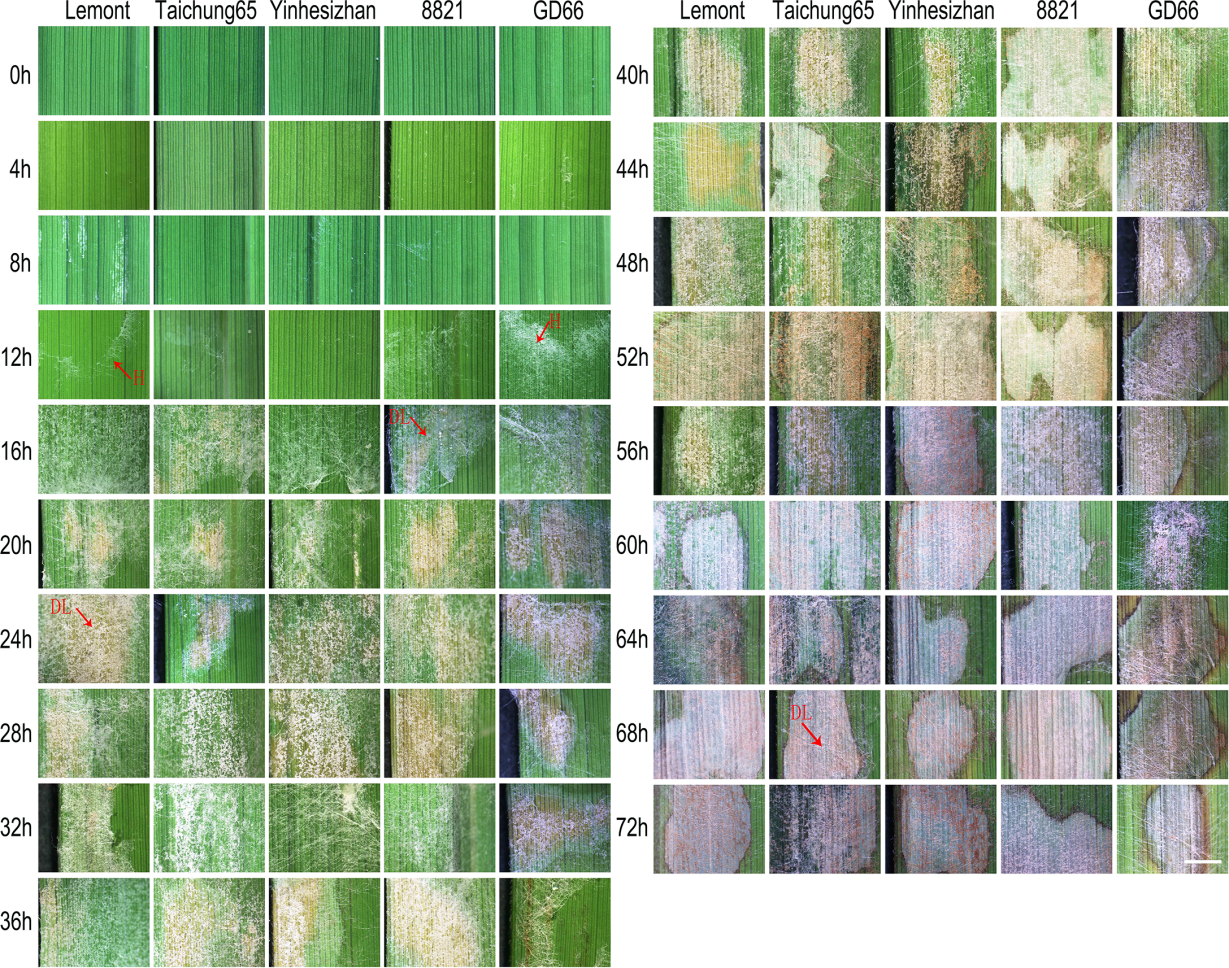
Sheath blight lesion development on rice leaf of five genotypes at every four h after *in vitro* inoculation observed under a stereoscopic microscope. Scale bar = 2 mm. H, hyphae; DL, disease lesion.

Apparent lesions were not observed on leave surfaces of all genotypes from 0 to 12 hpi. Infection symptoms gradually arose at 16 hpi, and the leaf color started to turn yellow at the infection site. It can be easily found that the white hyphae covered the leaf surface at 16 hpi and formed a network structure. For most genotypes, the lesions stay yellow from 16 to 52 hpi and turned gray from 56 to 72 hpi. Typical sheath blight lesions usually accompanied with the gray color at 56 hpi for most rice genotypes. The GD66 was more sensitive in sheath blight reaction than the other four genotypes. Gray lesions were found in leaves of GD66 from 16 to 32 hpi, whereas the lesions of the other four genotypes were still yellow. Typical sheath blight lesions were found at 44 hpi for GD66, about 12 h much earlier than the other four genotypes (**Figure 1**).

### Lesion on sheath surface observed after *in vitro* inoculation

**Figure 2:**
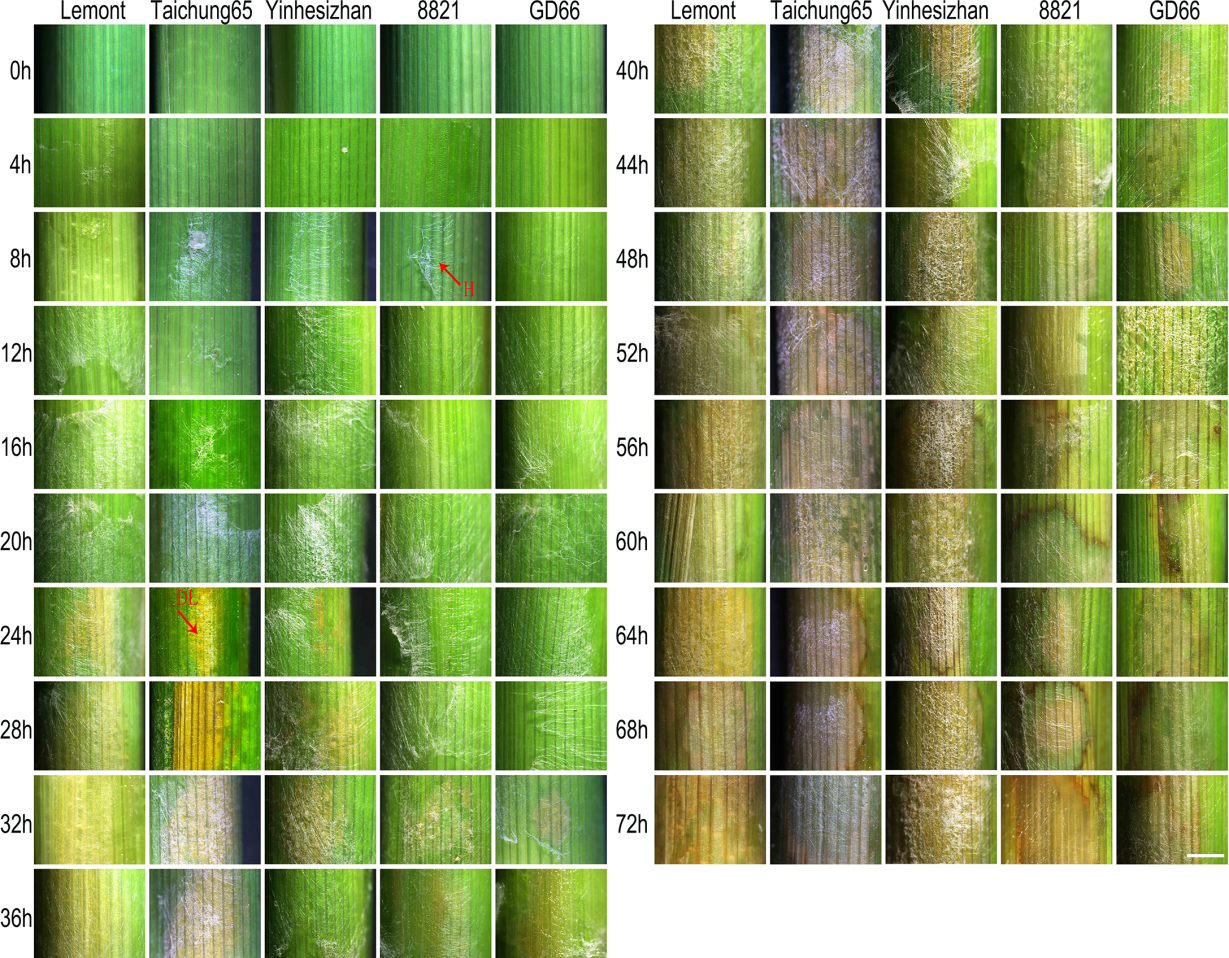
Sheath blight lesion development on rice sheath of five genotypes at every 4 h after *in vitro* inoculation observed under a stereoscopic microscope. Scale bar = 2 mm. H, hyphae; DL, disease lesion.

Although hyphae can be seen on sheath surface at 8 hpi, obvious symptoms were found at 24 hpi for Lemont, Taichung65, and Yinhesizhan and found at 32 hpi for GD66 and 8821. Typical sheath blight lesion appeared at 40 hpi for Taichung65, at 44 hpi for Lemont, at 48 hpi for Yinhesizhan, and at 60 hpi for GD66 and 8821, respectively (**Figure 2**).

### Developmental process of *R. solani* on rice leaf observed under a fluorescence microscope after *in vitro* inoculation

**Figure 3:**
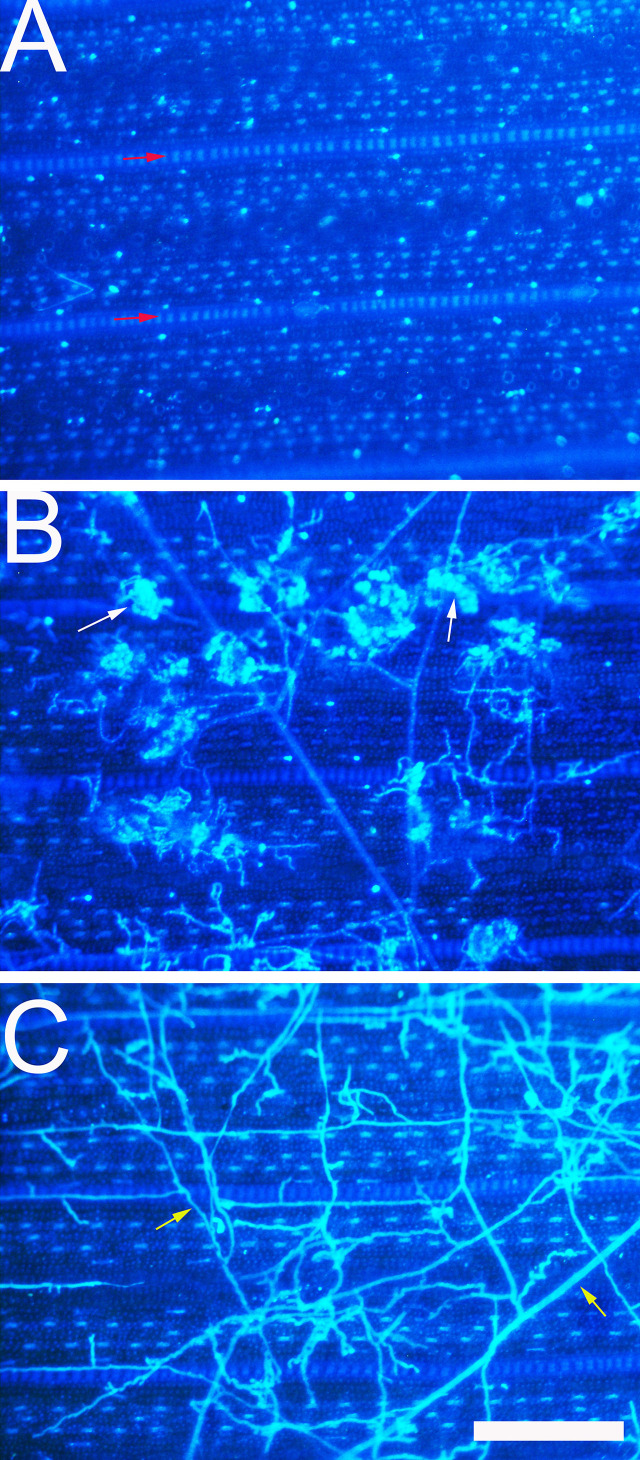
Development of *Rhizoctonia solani* hyphae and infection cushion on rice leaf observed under a fluorescence microscope. **(A)** The leaf surface of 8,821 at 0 hpi. Red arrow indicates vein. **(B)** The leaf surface of Lemont at 52 hpi. White arrow indicates the infection cushion of *R. solani.* **(C)** The leaf surface of Lemont at 72 hpi. Yellow arrow indicates hypha. Scale bar = 200 µm.

After stained with DAPI, the *R. solani* hyphae and infection cushions were clear. The bright color made them easy to be distinguished from rice leaf under a fluorescence microscope (**Figure 3**).

**Figure 4:**
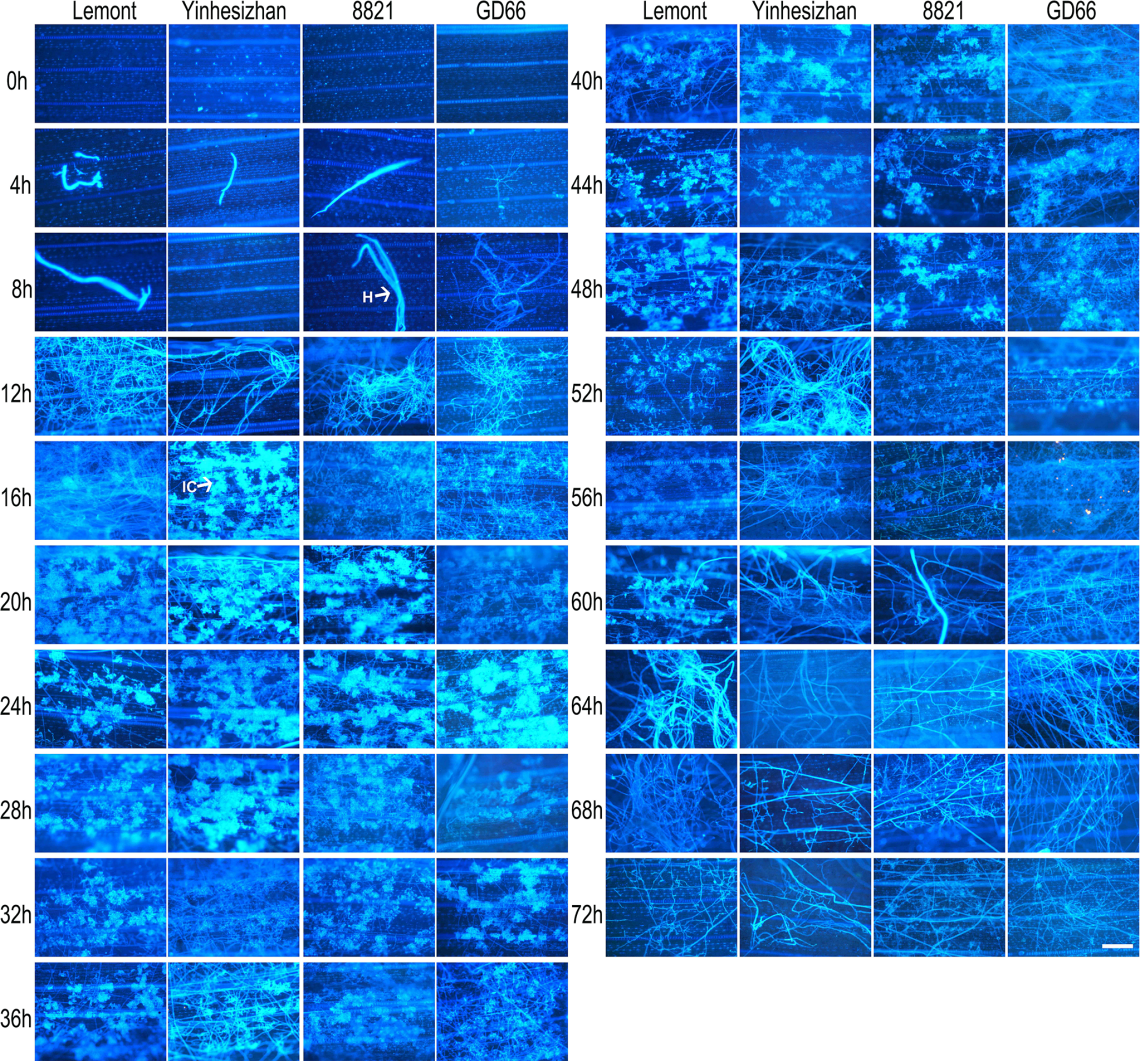
Development of *Rhizoctonia solani* hyphae and infection cushion on leaf of four rice genotypes at a serial time points after inoculation. The leaf samples were stained with DAPI and observed under a fluorescence microscope. Scale bar = 200 µm. H, hyphae; IC, infection cushions.

Hyphal growth was found on leaf surface at 4 hpi. From 4 to 8 hpi, the amount of hyphae was scarce. At 12 hpi, a large amount of hyphae covered the surface of rice leaves. A specialized penetration structure named infection cushion was clearly observed at 16 hpi. From 20 to 48 hpi, a large quantity of infection cushions occupied surface of leaves. The mount of infection cushions decreased gradually from 52 to 72 hpi (**Figure 4**).

### Developmental process of *R. solani* on rice sheath observed under a fluorescence microscope after *in vitro* inoculation

**Figure 5:**
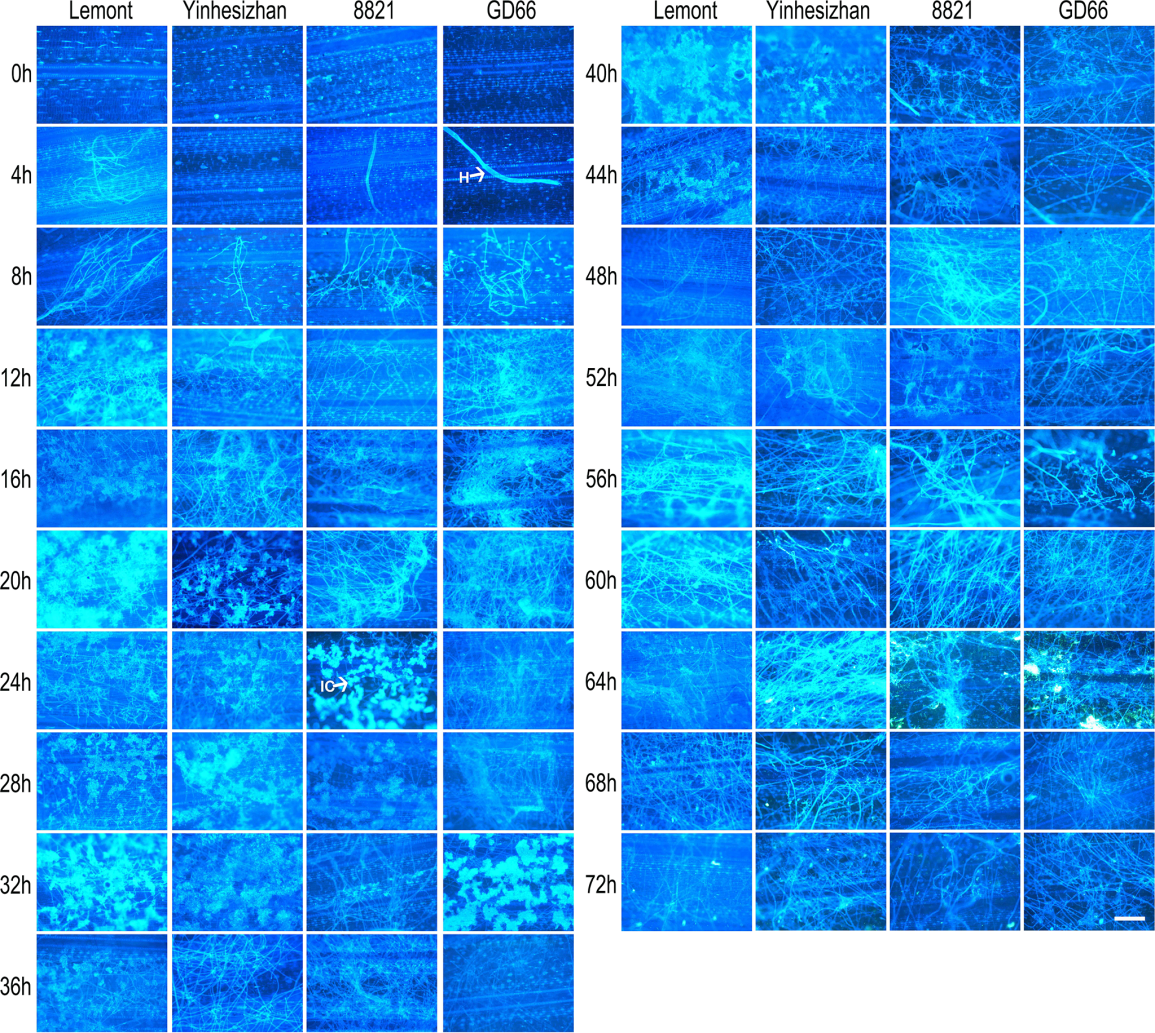
Development of *Rhizoctonia solani* hyphae and infection cushion on sheath of four rice genotypes at a serial time points after inoculation. The sheath samples were stained with DAPI and observed under a fluorescence microscope. Scale bar = 200 µm. H, hyphae; IC, infection cushions.

The development of hyphae and infection cushions on rice sheath was similar with those observed on rice leaf surface but with some differences. The infection cushions were first found at 12 hpi for Lemont, at 20 hpi for Yinhesizhan, at 24 hpi for 8821, and at 32 hpi for GD66, respectively. The infection cushion stayed a much shorter time on rice sheath surface compared with that on leaf surface (**Figure 5**). The infested structures spread along the edge of the lesion on the leaf, whereas there were only a few hyphae on the leaf sheath.

### Developmental process of *R. solani* on rice leaf and sheath observed by WE-CLSM

**Figure 6:**
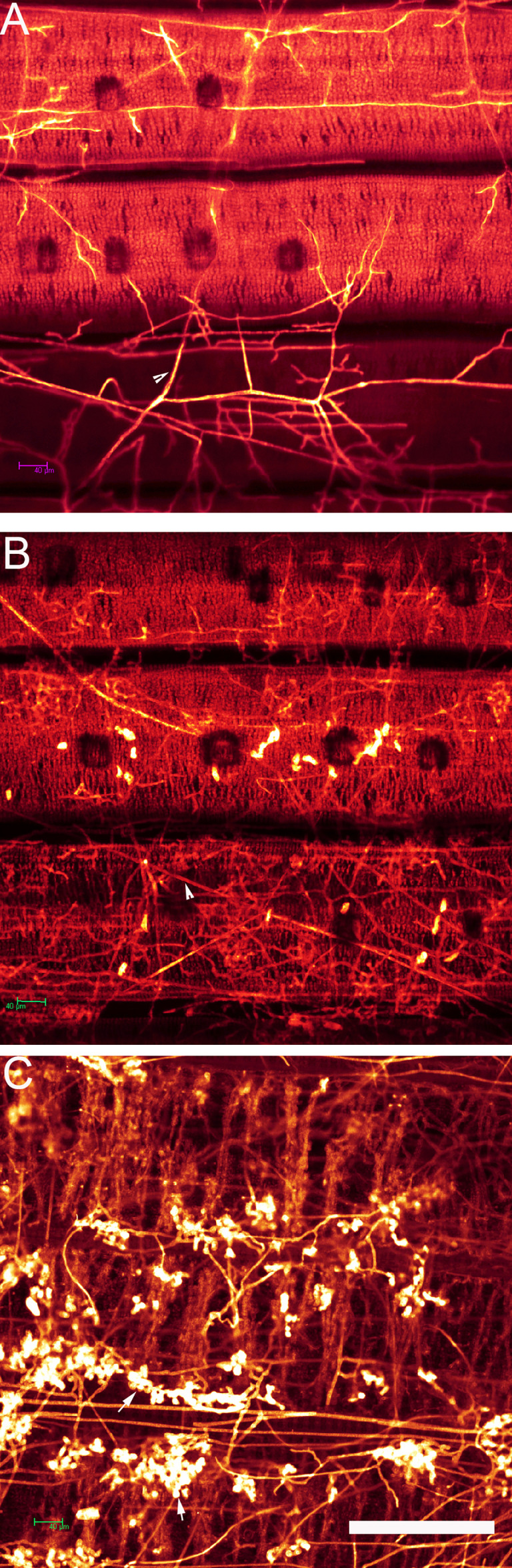
The whole-mount eosin B-staining confocal laser scanning microscopy developed in Xiangdong Liu’s laboratory was used for observing hyphal growth of *Rhizoctonia solani* on rice leaf. **(A)** Hyphae invading mesophyll cells on rice leaf at 12 h after *in vitro* inoculation. Arrowhead indicates the hypha. **(B)** Hyphae occupying a large number of spaces on rice leaf at 24 h after *in vitro* inoculation. Arrowhead indicates the hypha. **(C)** The mesophyll cells on rice leaf were almost destroyed and occupied by hyphae and infection cushions (arrows). Scale bar = 200 µm.

WE-CLSM, which was developed by our research team (Zeng et al., 2007), was first employed to observe the developmental process of hyphae and infection cushions on rice leaf and sheath surface by using confocal microscopy. The hyphae or infection cushions of *R. solani* showed yellow or brighter color, whereas the leaf or sheath showed red color after stained with eosin B (**Figure 6**).

**Figure 7:**
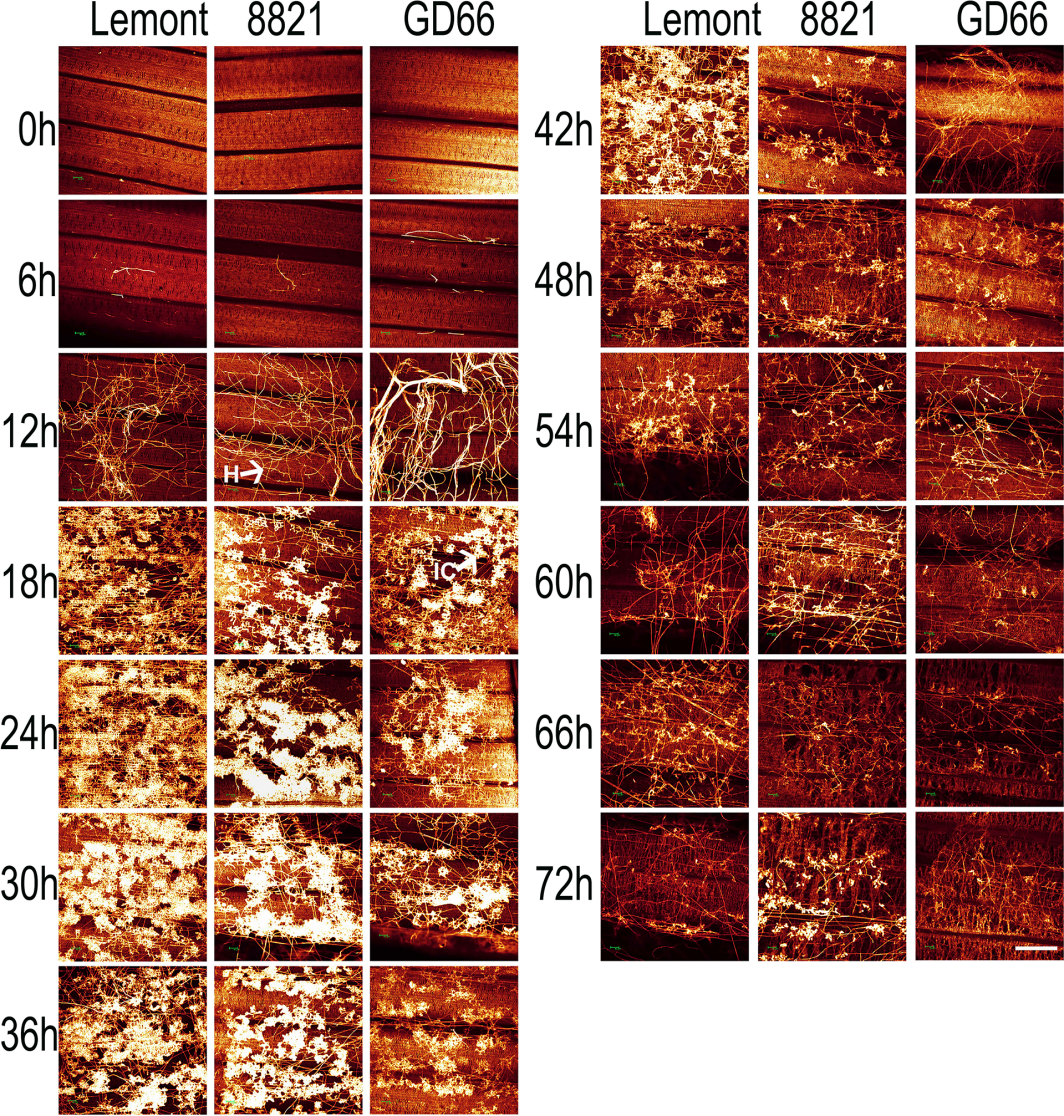
The whole-mount eosin B-staining confocal laser scanning microscopy showing developmental process of hyphae and infection cushions within rice leaf at a serial time point after inoculation. All images were obtained *via* the Leica SPE laser scanning confocal microscope. Excitation wavelength was 543 nm, and emission light was noticed between 550 and 630 nm. Scale bar = 200 µm. Three replications were applied for each sample. H, hyphae; IC, infection cushions.

**Figure 8:**
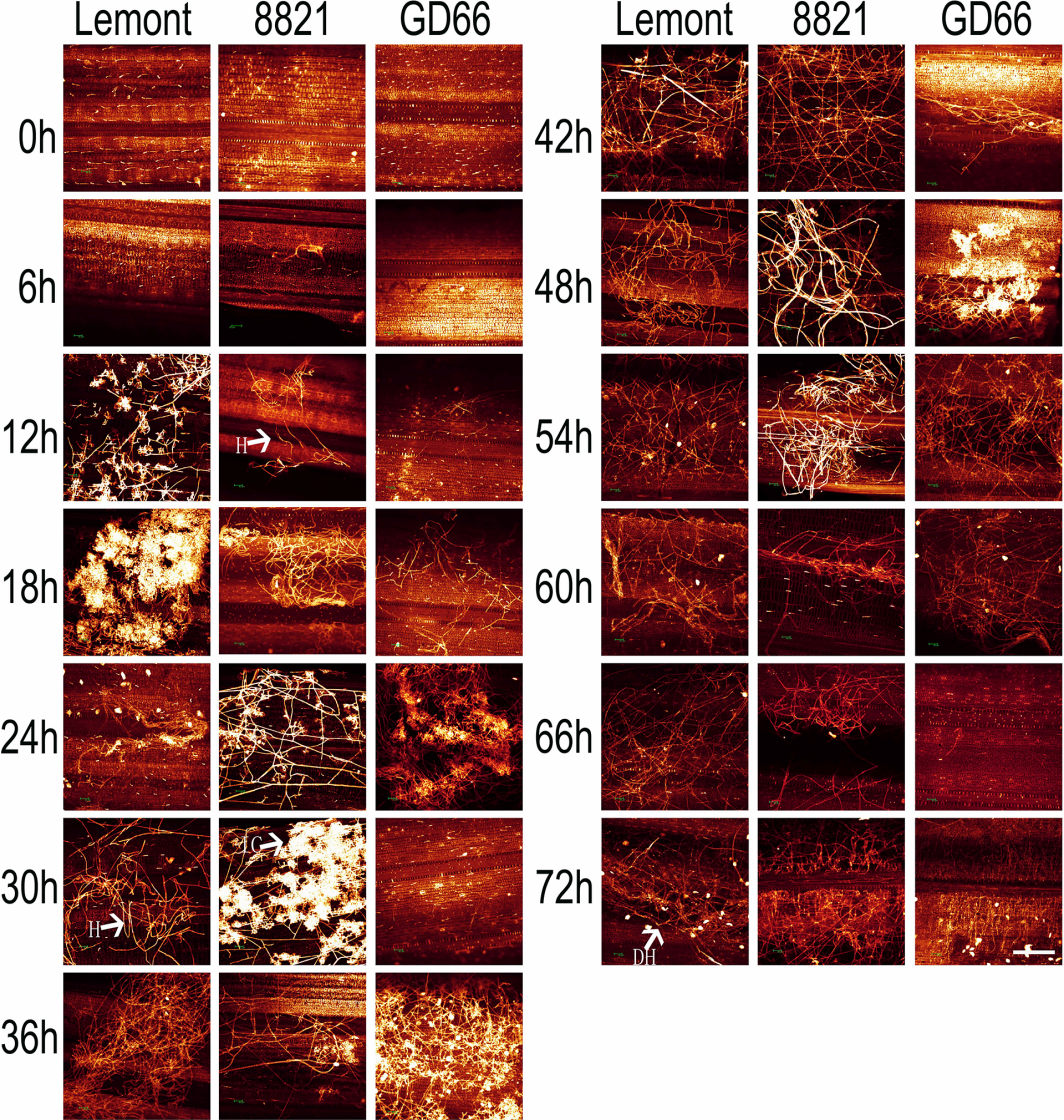
The whole-mount eosin B-staining confocal laser scanning microscopy showing developmental process of hyphae and infection cushions within rice sheath at a serial time point after inoculation. All images were obtained *via* the Leica SPE laser scanning confocal microscope. Excitation wavelength was 543 nm, and emission light was noticed between 550 and 630 nm. Scale bar = 200 µm. Three replications were applied for each sample. H, hyphae; IC, infection cushions. DH, decreased hyphae.

The hyphal growth was observed using WE-CLSM, and it was found that a large amount of hyphae covered leaf surface at 12 hpi. The infection cushions were clearly seen from 18 to 36 hpi, and they decreased gradually from 42 to 72 hpi (**Figure 7**). The situation was similar on sheath surface, but the infection cushions stayed a much shorter time on sheath surface than on leaf surface (**Figure 8**).

**Figure 9:**
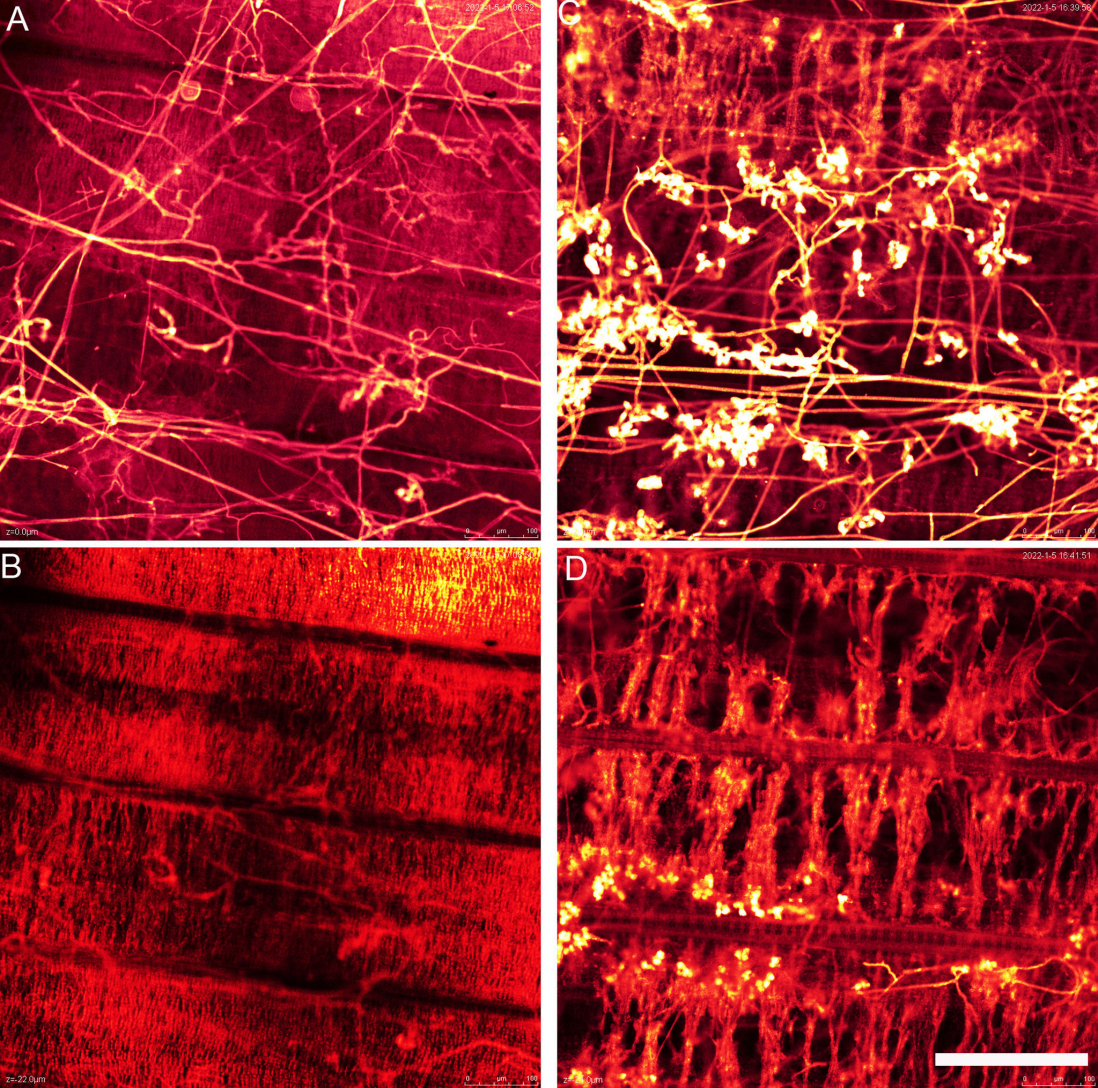
The hyphae above **(A)** and beneath **(B)** the GD66 leaf surface at 72 hpi as demonstrated by whole-mount eosin B-staining confocal laser scanning microscopy (WE-CLSM). **(B)** Twenty-two micrometers beneath GD66 leaf surface at 72 hpi. The infection cushions above **(C)** and beneath **(D)** the 8,821 leaf surface at 72 hpi as demonstrated by WE-CLSM. **(D)** Twenty-one micrometers beneath 8,821 leaf surface at 72 hpi. Scale bar = 200 µm.

WE-CLSM can show not only the leaf or sheath surface but also different layers inside leaf or sheath. There were a large amount of hyphae found on the leaf surface of GD66 at 72 hpi (**Figure 9**). However, only a few hyphae were found invading GD66 mesophyll cells at 22 µm beneath leaf surface (**Figure 9**). At 21 µm beneath leaf surface, the amount of infection cushions decreased dramatically compared with that on the leaf surface (**Figures 9C**).

Interestingly, *R. solani* could not only colonize on the abaxial surfaces of leaf sheath but also invade the paraxial side of the leaf sheath, suggesting that the hypha could pass through the surface of leaf sheath and move to the paraxial side and then colonize on both sides of leaf sheath. Cytological observation using semi-thin sections demonstrated this result (**Figure S5**). The observation in this study shows that the detailed positions where *R. solani* invading rice sheath were randomly distributed. WE-CLSM observation displayed that the *R. solani* could colonize on the paraxial side of leaf; however, it was not found on the abaxial side of the leaf, suggesting that the *R. solani* may have different infection mechanism between leaf sheath and leaf.

### Statistical analysis of RNA-seq results at different time points after *R. solani* inoculation

To analyze the transcriptional response to the *R. solani* infection in rice, leaf sheaths were inoculated with ZJ03; RNA-seq analysis was performed on samples at 0, 24, and 48 hpi; and each time point was repeated thrice with a total of 18 samples. An overview of the sequencing and mapping results is shown in **Supplementary Table S2**. A total of 49.2-GB raw data were obtained from 18 samples. An average of 44,355,838 reads was obtained for each sample, with a Q_30_ quality score ≥ 93.55%. Filtered reads were aligned with the rice genome (Japanese Rice MSU_v7.0; 62,120 transcripts), which was obtained from the Genome databases for Plant Biology (https://plantbiology.aspb.org), resulting in 83.74% mapping percentage, indicating a high quality of the data.

### Differential gene analysis in response to *R. solani* infection

**Figure 10:**
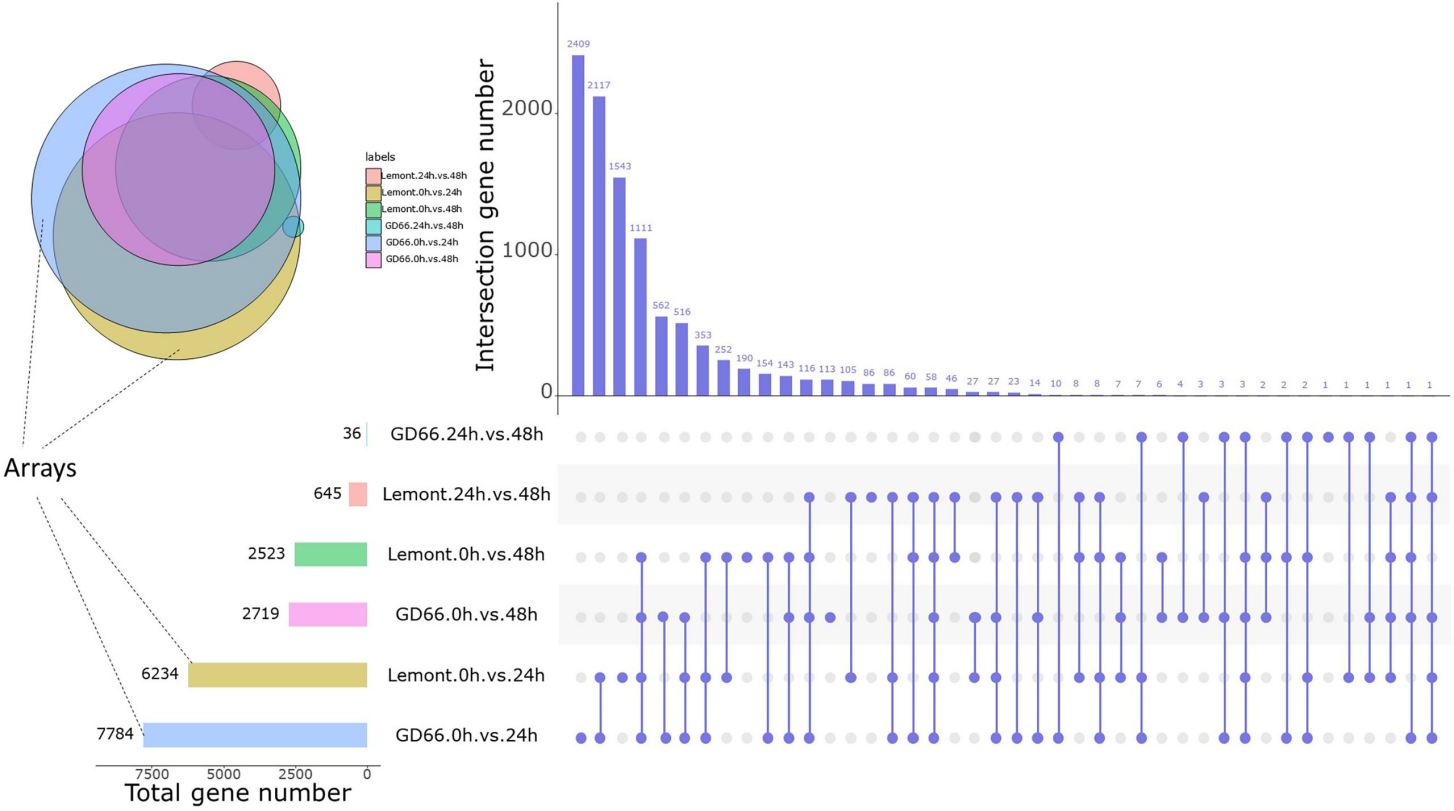
DEGs at three time points after inoculation between Lemont and GD66. The UpSet plot was used to visualize the total size and overlaps of DEGs in various data sets. The colorful bar charts indicate DEGs under single pairwise comparison; the left side of x-axis represents the number of DEGs. Column charts indicate DEGs under single or multiple comparisons. On the right side of x-axis, slate blue dots represent specific DEGs of a single comparison, slate blue lines connected by dots represent intersection DEGs of multiple comparisons, and the y-axis represents the number of DEGs corresponding to them. Colored circles indicate the Venn diagram of multiple comparisons of DEGs, with the same color corresponding to the colored bar chart on the left.

A total of 6,234 and 2,523 DEGs were detected for Lemont at 24 and 48 hpi, respectively. In addition, 7,784 and 2,719 DEGs were detected for GD66 at 24 and 48 hpi, respectively (**Figure 10**). This indicated that the gene expression reached maximum extent at 24 hpi and then descended at that time for both genotypes.

The number of DEGs was different between Lemont and GD66, and the upregulated genes exceeded those of the downregulated genes for both genotypes at different time points after inoculation: At 24 hpi, 6,234 DEGs were found in Lemont, with 3,244 upregulated genes and 2,990 downregulated genes. A total of 7,784 DEGs were identified in GD66, of which 4,177 were upregulated and 3607 were downregulated at 24 hpi. The same trend was found at 48 hpi (**Table S3**). It suggested that the resistance genotype GD66 recruited more genes than the susceptible genotype Lemont to fight against sheath blight pathogen.

**Figure 11:**
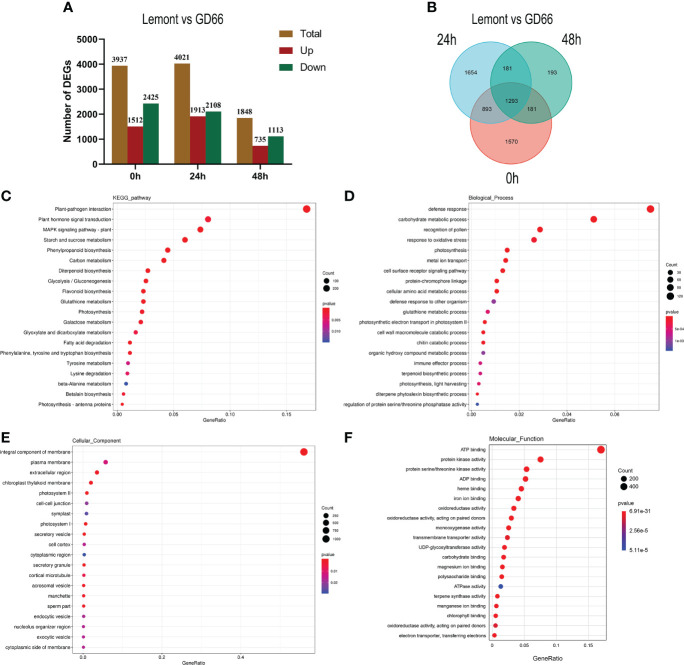
Differentially expressed genes (DEG) and Kyoto Encyclopedia of Genes and Genomes (KEGG) and GO analysis. **(A)** Differentially expressed genes (DEG) between Lemont and GD66 detected at different time points after inoculation. Red color indicates upregulated genes, and green color indicates downregulated genes. **(B)** Venn diagram showing overlapping DEGs at three time points. **(C)** The KEGG pathway enrichment analysis of the DEGs between Lemont and GD66 at three time points. **(D–F)** Visualization of GO enrichment terms for total DEGs in Biological_Process, Cellular Component, and Molecular_Function Ontology, respectively.

We compared the sheath blight–resistant genotype GD66 and the susceptible genotype Lemont and found that there were 3,937, 4,021, and 1,848 DEGs at 0, 24, and 48 h, respectively (**Figure 11**). The results showed that there were great differences on gene expression before and after *R. solani* infection. The DEGs were also illustrated in a Venn diagram. It was clear that 1,293 DEGs were found to be continuously response in all time points (**Figure 11**).

### Kyoto Encyclopedia of Genes and Genomes pathways and Gene Ontology annotation of the differentially expressed genes

A total of 5,965 DEGs were detected between Lemont and GD66 at different time points after inoculation (**Figure 11**). Kyoto Encyclopedia of Genes and Genomes (KEGG) annotation revealed that the 5,965 DEGs were mapped to 132 KEGG pathways (*p <*0.05, **Table S4**). The highest enriched pathway was plant–pathogen interactions (KO04626), followed by plant hormone signal transduction (KO 04075), mitogen-activated protein kinase (MAPK) signaling pathway plant (KO04016), starch and sucrose metabolism (KO 00500), phenylpropanoid biosynthesis (KO 00940), and carbon metabolism (KO 01200) (**Figure 11**).

The 5,965 DEGs were annotated using GO database *via* the Blast2GO program (http://www.blast2go.com/). The most significantly enriched GO terms include the following: “defense response”, “carbohydrate metabolic process”, “recognition of pollen”, and “response to oxidative stress” in Biological Process Ontology (**Figure 11**, **Table S5**); “integral component of membrane”, “plasma membrane”, “extracellular region”, and “chloroplast thylakoid membrane” in Cellular Component Ontology (**Figure 11**, **Table S6**); “ATP binding”, “protein kinase activity”, “protein serine/threonine kinase activity”, and “ADP binding” in Molecular Function Ontology (**Figure 11**, **Table S7**). These results suggest that the complex molecular defense reaction was triggered in rice after *R. solani* invasion.

### MapMan analysis

**Figure 12:**
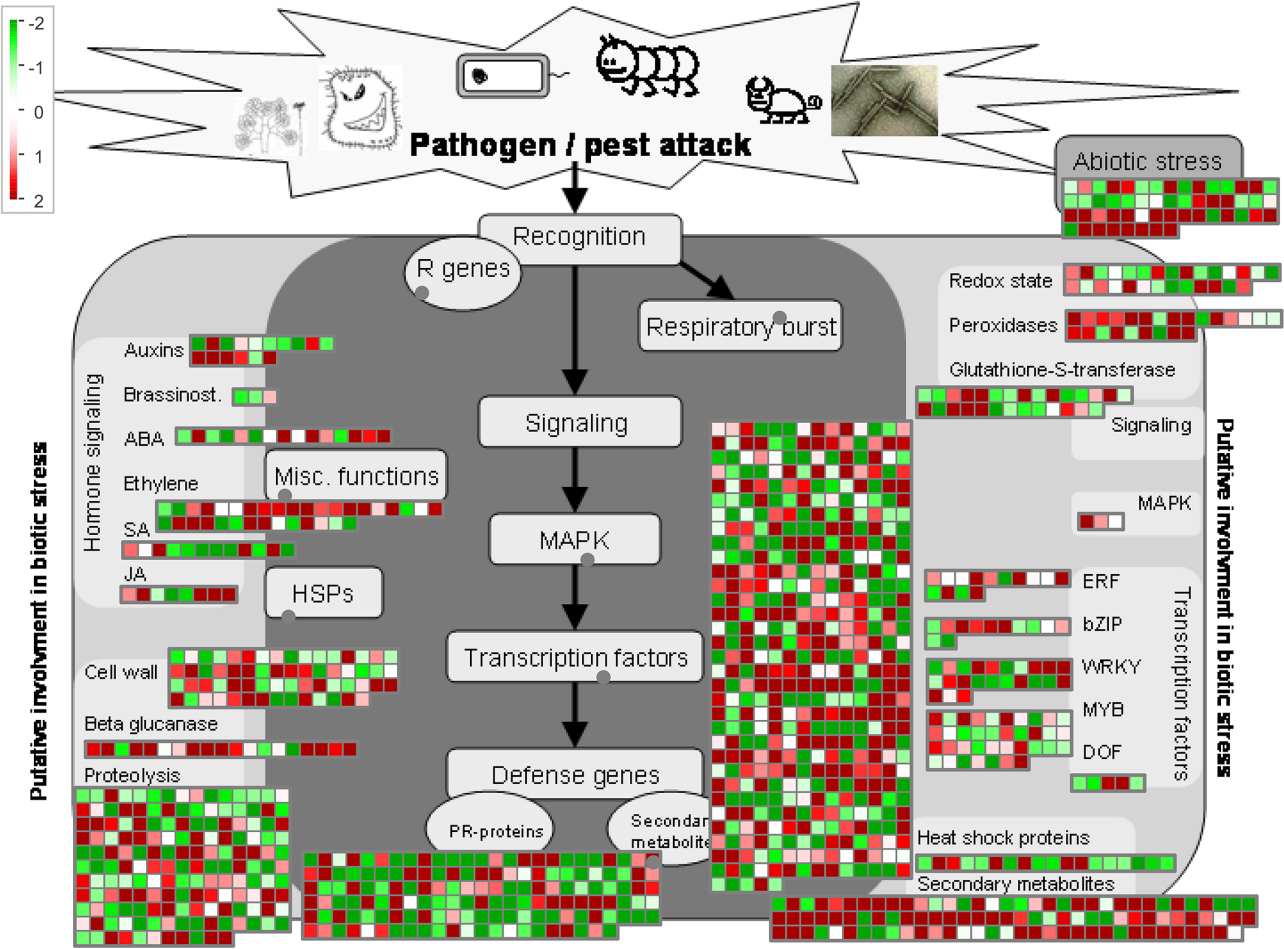
MapMan analysis of the DEGs between GD66 and Lemont at 24 hpi based on the log_2_FC value. Red, upregulate; green, downregulate.

Because the DEGs reached maximum quantity at 24 hpi, we compared the DEGs between GD66 and Lemont at 24 hpi. The MapMan analysis showed that 392 DEGs were involved in the signaling regulation pathway. A total of 161, 147, 100, 62, 53, 37, 34, 28, 28, 24, and 23 DEGs were participated in the proteolysis, PR-protein, secondary metabolites, cell wall–related, abiotic stress, MYB transcription factor, ethylene, glutathione-S-transferase, redox state, peroxidases, and WRKY regulatory pathways, respectively. In addition, 19, 18, 16, 15, 14, 12, 12, 8, 5, 3, and 3 DEGs were involved in the glucanase, heat shock protein, auxin, Abscisic acid (ABA), Ethylene-responsive factor (ERF), salicylic acid, bZIP transcription factor, jasmonate, DNA binding with one finger (DOF), zinc finger protein, mitogen-activated protein (MAP) kinase, and brassinosteroid regulatory pathways, respectively (**Figure 12**, **Table S8**).

### Validation of RNA-seq results using qRT-PCR

**Figure 13:**
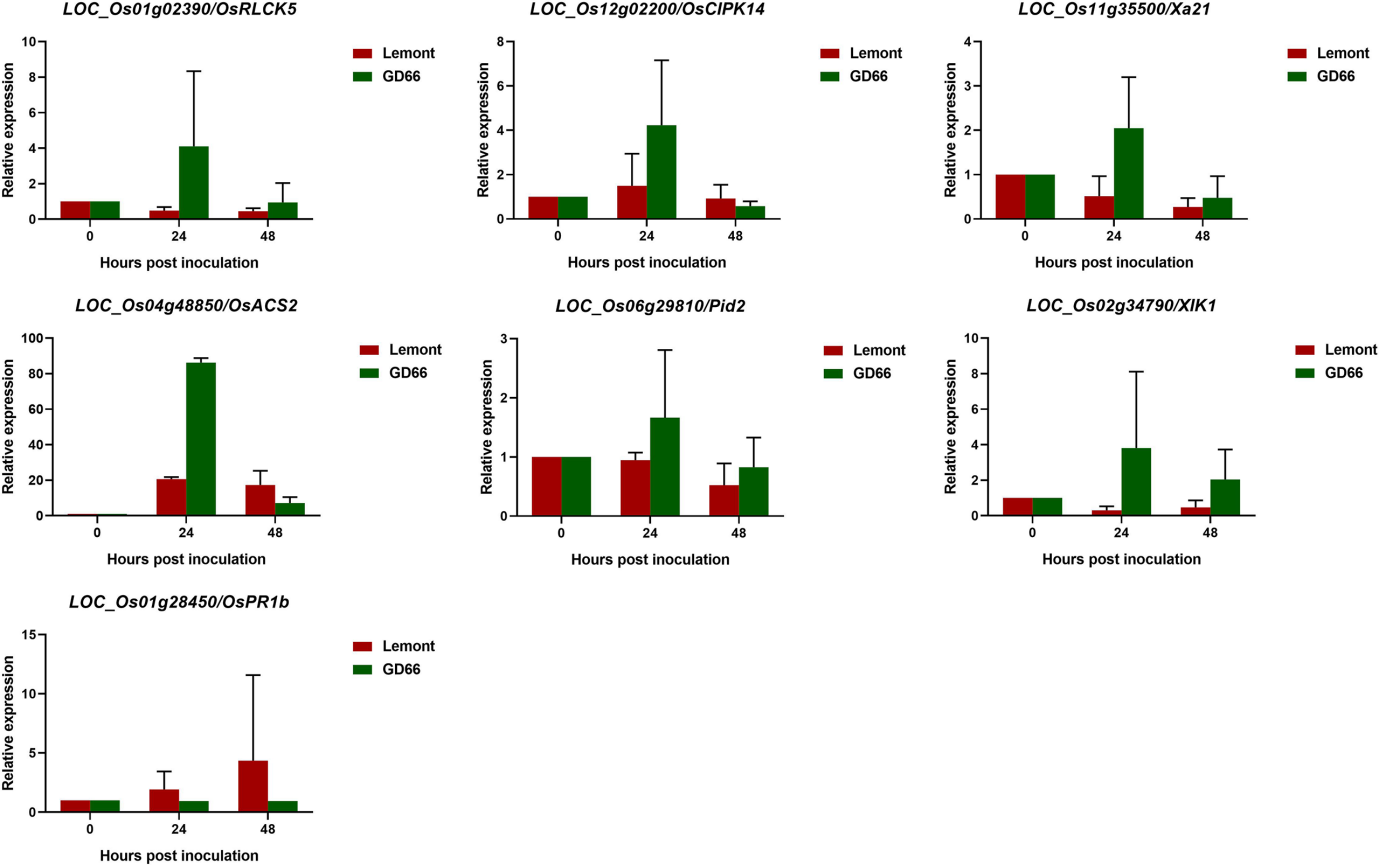
Quantitative real-time PCR analysis of seven rice genes.

The RNA-seq data were further validated by qRT-PCR analysis. Seven rice genes that have been reported to be associated with disease resistance were chosen for qRT-PCR analysis. Melting curves of qPCR products showed a unique peak for all genes, suggesting a good specificity of the primers (Pictures not shown). Rice *ubiquitin* gene was used as the internal control, and the *C*t values were normalized. The relative expression levels of the seven genes between Lemont and GD66 at different time points after inoculation were calculated. The qRT-PCR results showed the same trends with the RNA-seq data (**Figure 13**, **Table S9**), suggesting that the Illumina sequencing data were good and reliable. The qRT-PCR results confirmed that *OsRLCK5*, *OsCIPK14*, *Xa21*, *OsACS2*, *Pid2*, and *XIK1* had higher expression in the resistant genotype than the susceptible genotype, whereas *OsPR1b* had lower expression.

## Discussion

### WE-CLSM is a wonderful method for observing hyphal development in rice leaf or leaf sheath after *R. solani* infection

The WE-CLSM was first developed in 2003 and named as “WE-CLSM” in 2007 in our research team laboratory for studying rice embryo sac and other rice tissues (Zhang et al., 2003; Zeng et al., 2007; Li et al., 2017). For the first time, we used this technique to study rice leaf or sheath after sheath blight infection in this study. The results showed that it was powerful for observing hyphal growth within rice leaf or sheath: The hyphae or infection cushions are in yellow or brighter color under the red mesophyll cell background, which makes them easy to be distinguished from the rice mesophyll cells under the confocal microscope (**Figure 9**). WE-CLSM has the advantage of displaying detailed information at any layers beneath the leaf epidermal cells without needing sections, which makes it more convenient than the conventional sectioning method. It was the first time to find that *R. solani* could not only colonize on the abaxial surfaces of leaf sheath but also invade the paraxial side of the leaf sheath. However, the *R. solani* could only colonize on the one side of leaf. The infested structures spread along the edge of the lesion on the leaf, whereas there were only a few hyphae found on the surface of leaf sheath. This could be explained by the results of plastic semi-thin sections and WE-CLSM observation: The hyphae and infested structures did not traverse the leaf, and infestation pads destroyed only the outer cell wall structure of the leaf. The hyphae formed clustered rod-like polymers that absorbed nutrients from the mesophyll cells inside the leaves. The hyphae or infested structures may not penetrate the tight tissue structure in the leaves, so it could only colonize on the paraxial side of leaf. There was a large amount of air in the cavity of the leaf sheath, and hyphae can spread and move freely. Moreover, the parenchyma cells in the inner layer of the leaf sheath were relatively easy to be destroyed. Therefore, the pathogen can penetrate and colonize on both sides of the leaf sheath. After entering the inner part and colonizing on the paraxial side of the leaf sheath, the infested structures decreased on the abaxial side of the leaf sheath. In addition, GD66 is a highly resistant genotype. In fact, GD66 is the most resistant genotype among 211 genotypes tested in six environments from 2012 to 2016 (Zeng et al., 2017). However, the lesion developed much earlier on leaves of GD66 than the other four genotypes as demonstrated in **Figure 1**. It suggested that the mechanism of *R. solani*–infecting rice sheath was different from that of leaf.

In addition to WE-CLSM, we also used a fluorescence microscope to observe the hyphal growth on rice leaf and leaf sheath. Both methods let the hyphae and infection cushions of *R. solani* clearer and easy to be distinguished from the leaf cells. However, the fluorescence microscopy can only display the situation on leaf surface; it cannot see through the leaf epidermis cells and show what is beneath the surface. However, WE-CLSM can demonstrate detailed information at any sections beneath leaf or sheath surface.

Using both fluorescence and WE-CLSM, it was found that the amount of hyphae increased from 4 to 12 hpi on leaf surface. In addition, the infection cushions formed at about 16 hpi and maintained large quantities from 20 to 48 hpi and then decreased gradually from 52 to 72 hpi on leaf surface. The situation was similar on sheath surface, but the infection cushions stayed shorter time on sheath surface than on leaf surface. We also found that the amount of hyphae or infection cushions decreased dramatically at 20 µm beneath leaf surface at 72 hpi by using confocal microscopy. In addition, we observed that sheath blight pathogen can penetrate the upper epidermis cells of sheath and then pass through the mesophyll cell and reach the lower epidermis cells (picture not shown). This suggested that the pathogen can harm rice sheath at two directions: (1) from the upper epidermis to the lower epidermis by entering the mesophyll cells at vertical direction and (2) occupying more area on sheath surfaces at horizontal direction.

### DEGs between sheath blight–resistant and sheath blight–susceptible genotypes

In recent years, the advancement of next-generation sequencing technique has greatly promoted genome and transcriptome research in different organisms (Wang et al., 2009; Jain, 2012). RNA-seq, a sequencing technique, has been widely used in studying plant pathogenesis, host-defense responses, and mechanisms of plant–pathogen interactions (Kawahara et al., 2012; Lai and Mengiste, 2013; Okubara et al., 2014). To decipher the differential gene expression responses during the *R. solani* infection, a time-series RNA-seq analysis (i.e., 0, 24, and 48 h) was performed, and a total of 7,950 and 7,080 DEGs in GD66 and Lemont were identified, respectively. Our results are consistent with those by Samal et al. (2022), who detected more DEGs in the resistant genotype CR1014 than that in the susceptible genotype Swarna-Sub1. Similarly, stronger and earlier responses to pathogen invasion at the transcriptome level have been found in the resistant grapevine specie *Vitis riparia* infected with *Plasmopara viticola* (Polesani et al., 2010).

In the present study, a total of 3,937 DEGs were detected between Lemont and GD66 before pathogen inoculation, suggesting that these two genotypes had great differences in genetic background. The fact that there were more DEGs in GD66 than that in Lemont at both 24 and 48 hpi indicated that the resistant genotype GD66 recruited more genes than the susceptible genotype Lemont in fighting against the pathogen. Our results are different from some previous reports that more DEGs were found in sheath blight–susceptible cultivars than that in sheath blight–resistant cultivars (Zhang et al., 2017; Shi et al., 2020; Yang et al., 2022). It means that the GD66 may have different mechanism than other resistant cultivars during rice–*R. solani* interaction.

A comparison between the resistance variety Teqing and the susceptible variety Lemont showed that the DEGs mainly concentrated in the early stages after *R. solani* AG1 IA infection (Zhang et al., 2017). A similar result was also found in the present study. We found that the DEGs reached maximum extent at 24 hpi and then decreased at 48 hpi. It suggested that the changes in *R. solani*–induced gene expression were dynamic. This result is consistent with reports on different plants and emphasizes the importance of time series analyses in understanding *R. solani*–plant interaction (Moore et al., 2011; Windram et al., 2012; Zhu et al., 2016).

### Genetic regulatory pathways involved in *R. solani*–rice interaction

Transcriptome analysis revealed complex gene regulatory network during *R. solani* infection in the present study. KEGG analysis showed that the DEGs were significantly enriched in pathways such as “plant–pathogen interactions”, ‘plant hormone signal transduction”, “MAPK signaling pathway”, “starch and sucrose metabolism”, and “phenylpropanoid biosynthesis”. Similar regulatory pathways have also been reported in some previous studies (Zhang et al., 2017; Yuan et al., 2018; Das et al., 2022). GO enrichment analysis showed that three important processes were involved during pathogen infection: (1) defense response, (2) carbohydrate metabolic process, and (3) oxidative stress response (**Figure 11**).

It has been reported that *OsRLCK5* interacted with *OsGRX20* and positively regulated rice resistance to *R. solani* (Wang et al., 2021). We found a higher expression of *OsRLCK5* in the resistant genotype GD66 at both 24 and 48 hpi. The expression levels of *OsRLCK5* are low in the susceptible genotype Lemont as shown by qRT-PCR analysis (**Figure 13**). We speculate that a higher expression of *OsRLCK5* may contribute to the sheath blight resistance in GD66.

Rice blast is a serious rice disease. Our study showed that some blast resistance genes may participate in the sheath blight resistance. *Pid2* encodes a receptor-like kinase protein, and it confers race-specific resistance to the *Magnaporthe grisea* strain, ZB15 (Chen et al., 2006). We found that the expression of *Pid2* was higher in GD66 than that in Lemont at both 24 and 48 hpi (**Figure 13**). It suggested that the *Pid2* may be related to sheath blight resistance. The *Pi9*, which encodes a nucleotide-binding site–leucine-rich repeat protein, is a broad-spectrum blast resistance gene in rice (Qu et al., 2006). We found that the expression of *Pi9* (*LOC_Os06g17900*) was higher in the resistant genotype GD66 than that in the susceptible genotype Lemont before or after inoculation (**Table S10**). This suggested that the *Pi9* may be related to the basic resistance of GD66. *OsCPK12* encodes a rice calcium-dependent protein kinase, and it negatively regulates rice blast resistance (Asano et al., 2012). In the present study, a lower expression level of *OsCPK12* in the resistance genotype GD66 and a higher expression in the susceptible genotype Lemont were detected. It suggested that the *OsCPK12* may negatively regulate sheath blight resistance.

It has been reported that OslecRK, a rice lectin receptor-like kinase, is important for resistance to rice blast and bacterial blight diseases (Cheng et al., 2013). In the present study, the expression of *OslecRK* (*LOC_Os04g12540*) in GD66 was twice as that in Lemont at 24 hpi (**Table S10**). Two ethylene biosynthetic genes, *OsACS1* and *OsACS2*, are induced by *M. oryzae* infection (Iwai et al., 2006). The expression of *OsACS2* (*LOC_Os04g48850*) in GD66 was about five times as that in Lemont at 24 hpi based on the RNA-seq data (**Table S9**) or qRT-PCR result (**Figure 13**). This indicates that the biosynthetic gene that contributes to basal resistance against *M. oryzae* also responds during *R. solani* challenging and may be related to the resistance in GD66.

The *WRKY* transcription factor has been proven to be regulated in plant defense responses, including positive and negative regulation of disease resistance (Pandey and Somssich, 2009). *OsWRKY24* encodes a protein that functions as a negative regulator of GA and ABA signaling (Zhang et al., 2009). We found that the *OsWRKY24* (*LOC_Os01g61080*) was differentially expressed in GD66 and Lemont at 24 hpi. The expression of *OsWRKY24* in GD66 was more than four times than that in Lemont (**Table S10**). It has been reported that overexpression of the *OsWRKY89* gene enhanced resistance to rice blast fungus and white-backed planthopper (Wang et al., 2007). Our results showed that the expression of *OsWRKY89* (*LOC_Os11g02520*) were lower in GD66 than that in Lemont before or after inoculation (**Table S10**). It seems that *OsWRKY89* may not be involved in the sheath blight resistance of GD66. In addition, the expression of *OsWRKY70* (*LOC_Os05g39720*) was significantly higher in GD66 than that in Lemont at 24 hpi (**Table S10**), suggesting that it may be related to sheath blight resistance in GD66.

*OsERF922* is a rice transcription factor that negatively regulates rice resistance to *M. oryzae* (Liu et al., 2012). In our study, the expression of *OsERF922* (*LOC_Os01g54890*) were lower in GD66 than that in Lemont before or after inoculation (**Table S10**), indicating that it may also negatively regulate sheath blight resistance in GD66.

It has been shown that *OsCIPK14/15* play crucial role in the microbe-associated molecular pattern–induced defense signaling pathway in rice (Kurusu et al., 2010). In our study, the expression level of *OsCIPK14* in GD66 was lower than that in Lemont before inoculation and at 48 hpi, but the expression of *OsCIPK14* in GD66 was increased dramatically compared with that of Lemont at 24 hpi (**Figure 13**, **Table S9**). Whether *OsCIPK14* participates in the regulation of sheath blight resistance needs to be clarified in the future.

*XIK1* encodes leucine-rich repeat receptor-like kinase and positively regulates *Xa21*-mediated rice bacterial blight disease resistance (Hu et al., 2015). Our RNA-seq result showed that the expression of *XIK1* (*LOC_Os02g34790*) was higher in GD66 than that in Lemont before or after *R. solani* inoculation (**Table S9**), suggesting that it may also involve in sheath blight resistance.

It has been reported that overexpression of *OsPGIP1* enhances rice resistance to sheath blight (Chen et al., 2016). However, our RNA-seq data showed that the expression level of *OsPGIP1* (*LOC_Os05g01380*) was lower in GD66 than that in Lemont before or after inoculation, suggesting that it may play an inessential role in the resistance genotype GD66.

A previous report showed that the levels of *OsPR1b* transcript was significantly upregulated in rice plant after infected by rice black-streaked dwarf virus (Lu et al., 2020). In the present study, we found that the expression of *OsPR1b* was significantly higher in Lemont than that in GD66 before or after inoculation (**Figure 13**, **Table S9**). How *OsPR1b* regulating sheath blight resistance needs to be characterized in the future.

Together, both RNA-seq and qRT-PCR results showed that genes participated in different regulatory pathways response to *R. solani* invasion: Genes relating to bacterial blight disease resistance (*Xa21*, *XIK1*), blast disease resistance (*Pid2*, *OsACS2*), rice black-streaked dwarf virus (*OsPR1b*), and other defense-related pathways altered significantly after *R. solani* infection.

We examined the DEGs between Lemont and GD66 and found that some of which contained typical NBS-LRR domain, such as *LOC_Os01g52330*, *LOC_Os12g13550*, and *LOC_Os12g10710* (**Table S8**). These three genes have not been characterized in previous reports. Because they expressed differentially between GD66 and Lemont, they might be associated with the sheath blight resistance. These candidate genes can be tested further.

### Insight in breeding a sheath blight–resistant rice cultivar

Genetic studies have shown that the rice sheath blight resistance is a typical quantitative trait controlled by many genes with minor effects. That means to breed a sheath blight–resistant cultivar, a lot of loci related to sheath blight resistance must be focused simultaneously. What genes should we focus? What is the target for selection in breeding? We recommend that the sheath blight–resistant genes characterized in previous studies should be the primary targets. We should also focus on genes that are participating in the sheath blight–resistant regulatory pathways identified by transcriptome analysis, although they may have not be cloned or characterized yet.

As we can learn from the present study, there were huge differences between the resistant genotype GD66 and the susceptible genotype Lemont at the transcriptome level. It is difficult to breed a sheath blight–resistant cultivar from a sheath blight–susceptible genetic background. In this study, we found that GD66 and 8821 are both superior genotypes with high resistance to sheath blight disease. If a sheath blight–susceptible cultivar was chosen as the parental parent in a breeding program, then we recommend using backcrossing to increase the sheath blight–resistant background of the maternal parent, such as 8821, to make sure that more sheath blight–resistant genes were selected in their descendants.

## Conclusion

A systematic cytological observation of developmental characteristics of *Rhizoctonia solani* hyphae and infection cushions on rice leaf and sheath displayed that the amount of hyphae increased dramatically on leaf and sheath surface at 12 hpi and that the infection cushions occurred and maintained at a huge number from 18 to 36 hpi, and then, the infction cushions disappeared gradually from 42 to 72 hpi. The *R. solani* could colonize on the abaxial sheath first, then penetrate the epidermis cell to inner part of sheath, and finally invade the paraxial side of leaf sheath. A different behavior of *R. solani* was found in rice leaf. RNA-seq analysis revealed that the resistant genotype GD66 recruited more than 5,000 DEGs to fight against the pathogen, which associated with some important resistant pathways, including bacterial blight disease resistance (*Xa21*, *XIK1*), blast disease resistance (*Pid2*, *OsACS2*), rice black-streaked dwarf virus (*OsPR1b*), and other defense-related genes. These results suggest that the WE-CLSM is a powerful technique in observing development of *R. solani* hyphae and infection cushions on leaf or sheath surface, and this process was associated with many resistance gene expressions simultaneously.

## Data availability statement

The data presented in the study are deposited in the NCBI SRA repository, accession number PRJNA886841.

## Author contributions

SL: Conceptualization, methodology, investigation, and data curation, writing—original draft, and writing—review and editing. TW: Investigation, data curation, and supervision. GM: Investigation and supervision. JL: Investigation, data curation, and supervision. DL: Investigation and supervision. XL: Conceptualization, methodology, resources, funding acquisition, and writing—review and editing. YZ: Conceptualization, methodology, resources, writing—original draft, and writing—review and editing. All authors contributed to the article and approved the submitted version.

## Funding

This work was supported by the Laboratory of Lingnan Modern Agriculture Project (NT2021001) and the Opening Foundation of State Key Laboratory for Conservation and Utilization of Subtropical Agro-Bioresources (202006).

## Acknowledgments

The authors thank Ms. Shuhong Yu and other laboratory members for assistance in experiment. We thank Wang Ling and Huang Shiwen at CNRRI for kindly providing the *R. solani* ZJ03 isolate.

## Conflict of interest

The authors declare that the research was conducted in the absence of any commercial or financial relationships that could be construed as a potential conflict of interest.

## Publisher’s note

All claims expressed in this article are solely those of the authors and do not necessarily represent those of their affiliated organizations, or those of the publisher, the editors and the reviewers. Any product that may be evaluated in this article, or claim that may be made by its manufacturer, is not guaranteed or endorsed by the publisher.
