# Supplementary material for: Cytological observation and transcriptome analysis reveal dynamic changes of Rhizoctonia solani colonization on leaf sheath and different genes recruited between the resistant and susceptible genotypes in rice
Source: Front Plant Sci. 2022 Nov 3;13:1055277. doi: 10.3389/fpls.2022.1055277 (PMC9669801; doi:10.3389/fpls.2022.1055277)
Supplement: Supplementary file 2 [file DataSheet_2.docx]

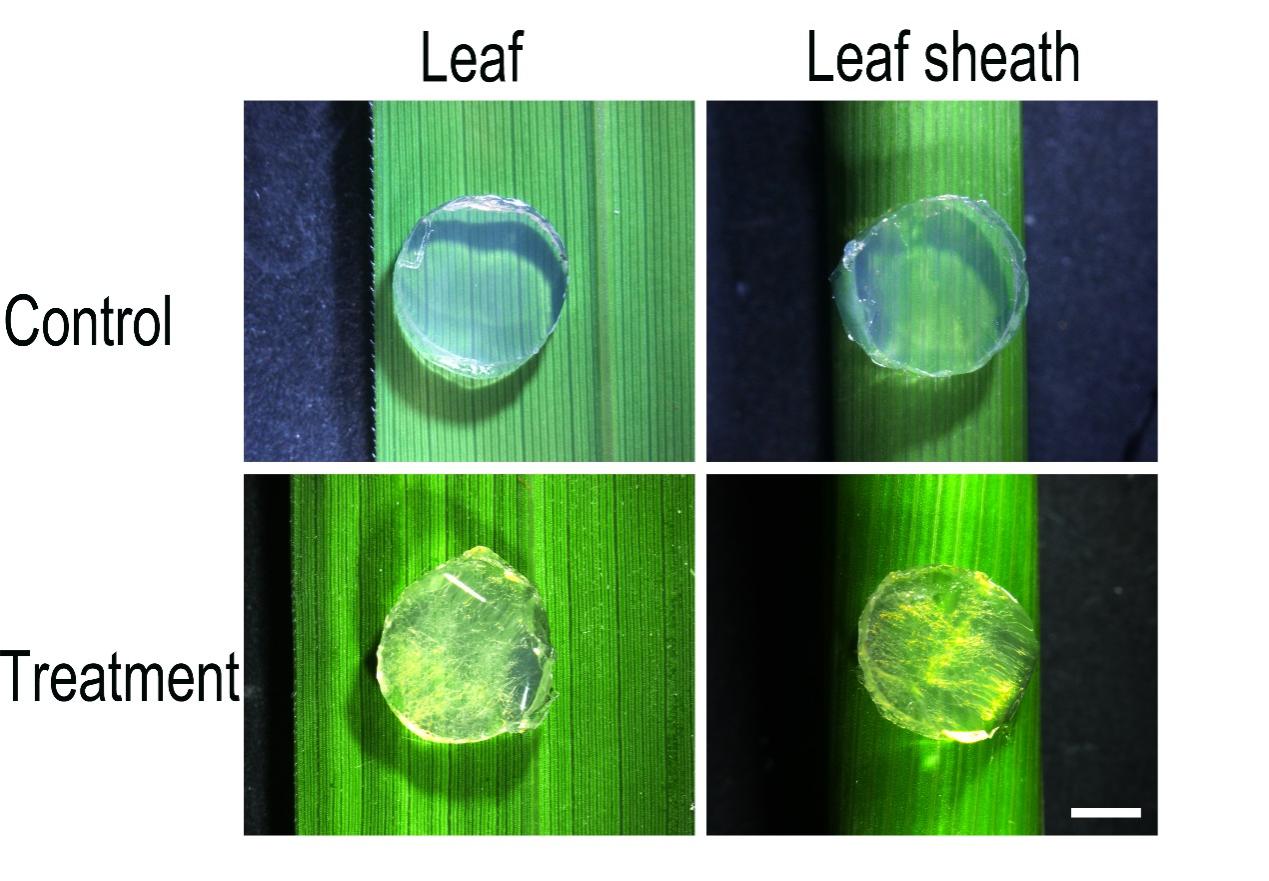
**Supplementary Figure 1.** A 5 mm diameter PDA medium covered with mycelia was placed on leaf or sheath surface for in vitro inoculation. PDA medium without mycelia was used as control. Scale bar = 2 mm.


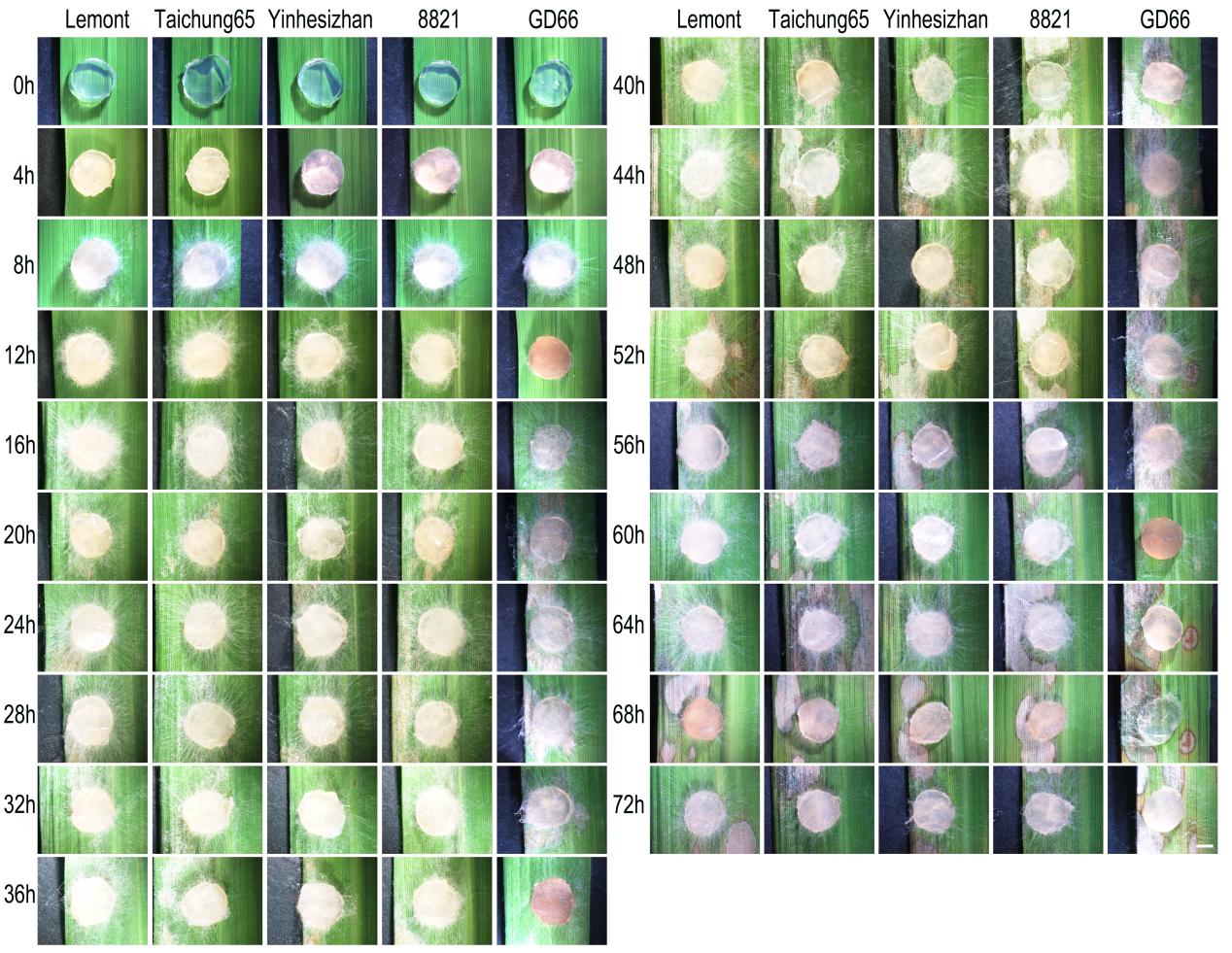


**Supplementary Figure 2.** In vitro inoculation on rice leaf surfaces of five rice genotypes. Scale bar = 2 mm.


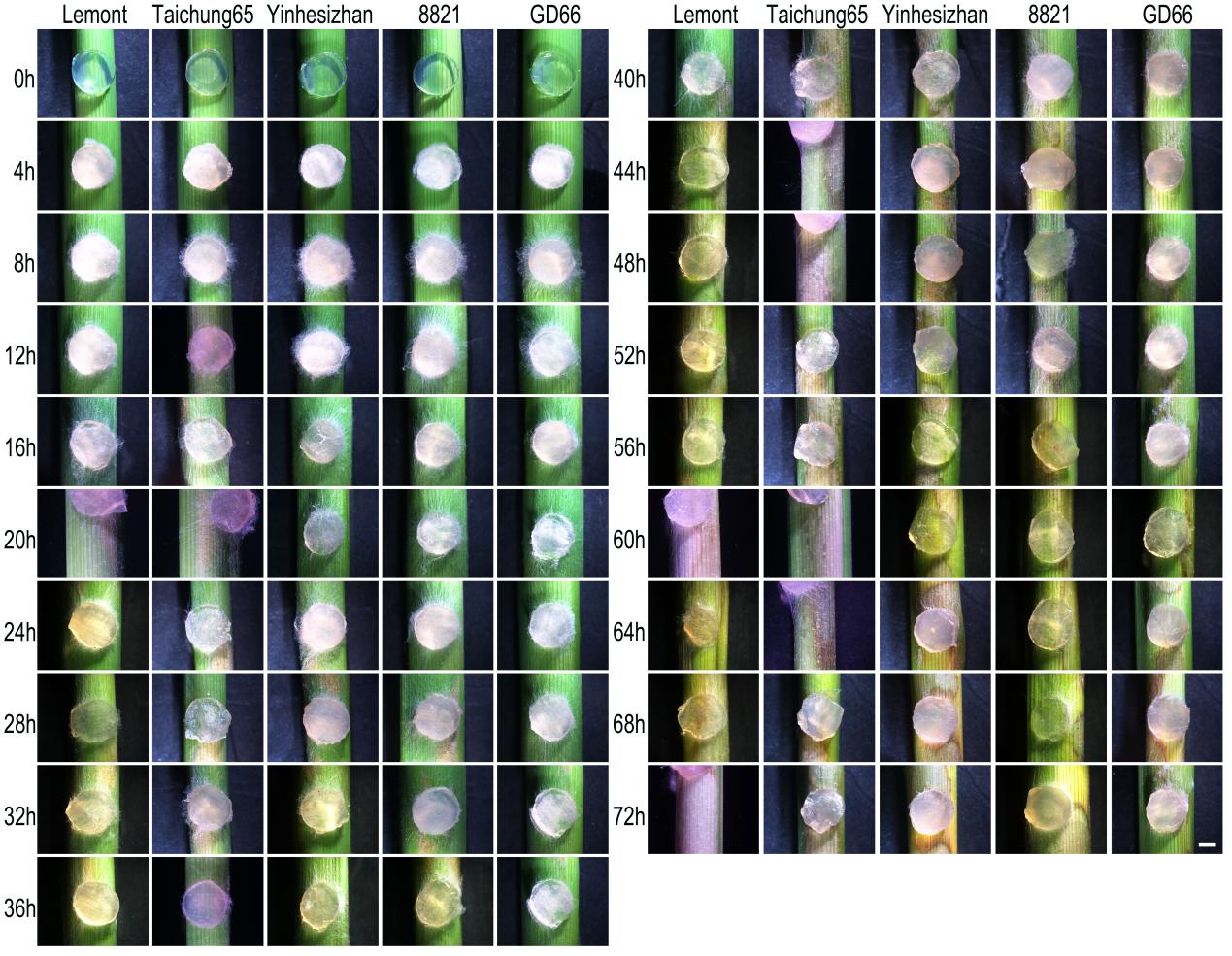


**Supplementary Figure 3.** In vitro inoculation on rice sheath surfaces of five rice genotypes. Scale bar = 2 mm.


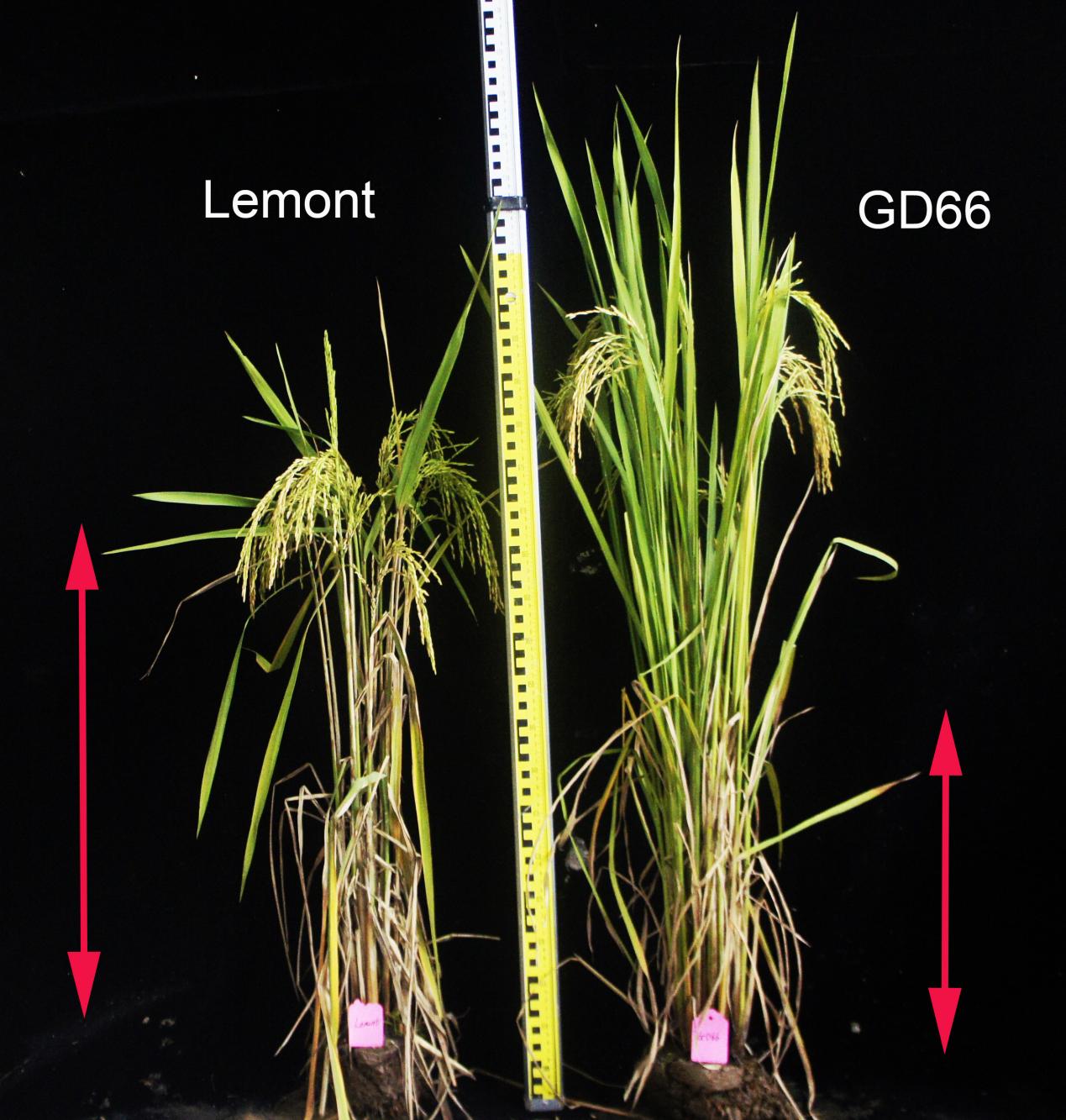


**Supplementary Figure 4.** Comparison of the sheath blight lesion length between a highly susceptible *japonica* cultivar Lemont and a highly resistant *indica* restorer line GD66.Photo was taken at 30 days after inoculation. Red double arrows indicate lesion lengths.


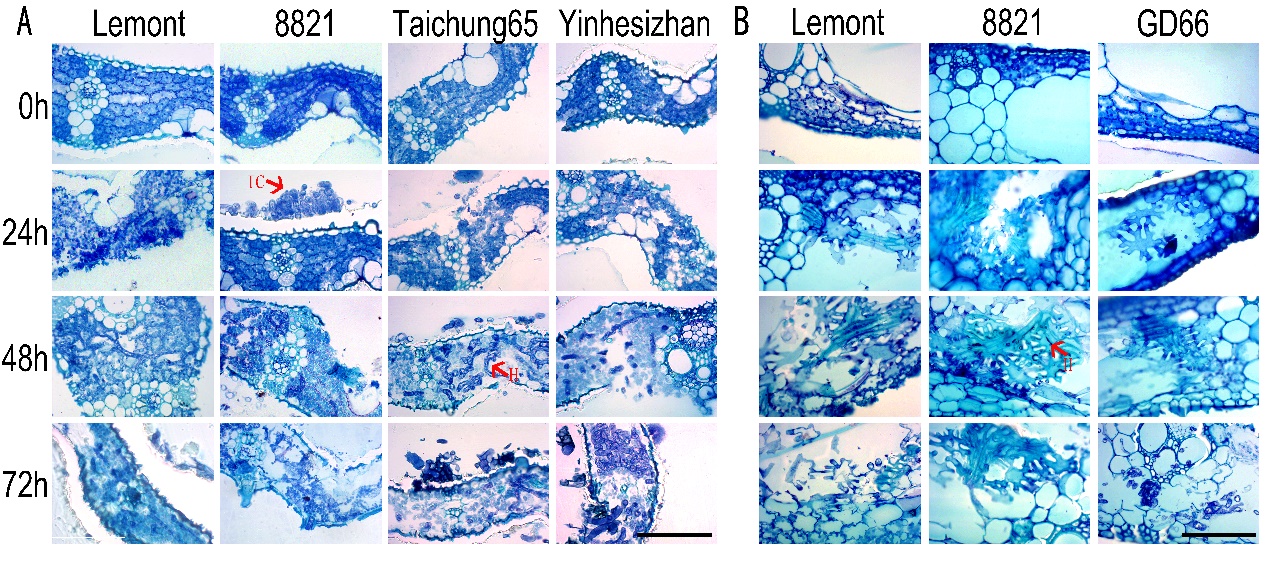


**Supplementary Figure 5.** Plastic semithin sections micrographs of different rice cultivars after infected by *Rhizoctonia solani.* The transverse sections stained by toluidine blue showed the morphology and structure of fungal hyphae inside the samples. (A) Morphology of the *Rhizoctonia solani* hyphae inside rice leaf. The hyphae formed a large number of clustered rod-like polymers. (B) The transverse sections showing fungal hyphae developed inside rice leaf sheath. The pathogen hyphae diffused and spread in the inner cavity of leaf sheath. Scale bars = 100 μm. All samples were collected from three independent plants in triplicate inoculation assays. H, hyphae; IC, infection cushions.
